# The Association Between Eating-Compensatory Behaviors and Affective Temperament in a Brazilian Population

**DOI:** 10.3389/fpsyg.2019.01924

**Published:** 2019-08-23

**Authors:** Sabrina Chapuis-de-Andrade, Carmen Moret-Tatay, Dalton Breno Costa, Francielle Abreu da Silva, Tatiana Quarti Irigaray, Diogo R. Lara

**Affiliations:** ^1^Pontifical Catholic University of Rio Grande do Sul, Porto Alegre, Brazil; ^2^Departamento de Neuropsicobiología, Metodología y Psicología Social, Facultad de Psicología, Universidad Católica de Valencia “San Vicente Mártir,” Sede de San Juan Bautista, Valencia, Spain; ^3^Federal University of Health Sciences of Porto Alegre, Porto Alegre, Brazil

**Keywords:** temperament, feeding and eating disorders, web-survey, personality, body image

## Abstract

Eating-compensatory behaviors are associated with biological and psychological complications, with high rates of morbidity and mortality. Different elements may contribute to the development of eating-compensatory behaviors, such as genetic, physiological, environmental, and temperamental factors. Therefore, the aim of this study was to examine the association between affective temperaments and eating-compensatory behaviors. A sample of 27,501 volunteers, between 18 and 55 years old, mean age 28.9 ± 8.7 years (69.6% women), were assessed by the Brazilian Internet Study on Temperament and Psychopathology (BRAINSTEP). The results showed that eating-compensatory behaviors were associated with distinctive affective temperaments. Cyclothymic types were more associated with eating-compensatory behaviors. The avoidant and irritable types presented lower percentages of eating-compensatory behaviors in women and men, respectively. In conclusion, this study highlighted that participants who adopted frequent eating-compensatory behaviors are more likely to have dysfunctional affective traits. Consequently, the affective temperaments should be considered as a strategy to build capacity for prevention, treatment, and care of eating-compensatory behaviors.

## Introduction

Eating disorders are critical public health problems. They are related to eating habits and behavior and they are associated with serious consequences for mental and physical health (American Psychiatric Association, 2013). According to the Fifth Edition of the Diagnostic and Statistical Manual of Mental Disorders (2013), the eating disorders with higher prevalence are anorexia nervosa, bulimia nervosa, and binge-eating disorder.

Previous studies show that in Latin America there is a lower prevalence of anorexia nervosa and a higher prevalence of bulimia nervosa and binge-eating disorder, compared to Western Europe or the United States (Kolar et al., 2016). In Brazil, the prevalence of bulimia nervosa is 2%, and binge-eating disorder is 4.7% in adults throughout life (Kessler et al., 2013).

According to other authors, there is a prevalence of 0.81% eating disorder diagnosis in the general population in Denmark, and 0.54% in Sweden (Mustelin et al., 2017). In Germany, eating disorders were reported for 1.5 and 5.9% of men and women, respectively (Hilbert et al., 2012). In Vietnam, this percentage reached 48.8% of university students (Ko et al., 2015). Regarding anorexia nervosa, a severe psychiatric illness, a study showed that it affects around 4% of girls in Europe (Keski-Rahkonen and Mustelin, 2016).

Importantly, even people who do not meet full criteria for an eating disorder may engage in some forms of eating-compensatory behaviors. Prolonged fasting, exhaustive physical exercise, vomiting, medication intake, diuretics, and laxatives are some examples of eating-compensatory behaviors and they are all risk factors for eating disorders. Of note, these behaviors are associated with biological and psychological complications, with high morbidity and mortality (Crow et al., 2008). Moreover, it is of great social interest due to the impact on the population, especially among young people and high financial costs to health and economy systems.

The frequent use of laxatives, auto-induced vomiting, and fasting for losing weight was reported by one-third to half of US girls (Cash and Smolak, 2011). Even older studies have already shown a high percentage of American teenagers, about 15% adopting eating-compensatory behaviors, as laxatives and diuretics intake (Phelps and Wilczenski, 1993). In Brazil, studies showed a high prevalence of abnormal eating behaviors and inappropriate methods of weight control in general population (Nunes et al., 2003; Chapuis-de-Andrade et al., 2017). Young women reported the use of laxatives (8.5%), restrictive diets (7.8%), misuse of appetite suppressants (5.1%), fasting (3.1%), misuse of diuretics (2.8%), and vomiting (1.4%) for losing weight (Nunes et al., 2003). Moreover, 15% of women with normal body mass index reported using diuretics and laxatives, and 12.2% reported vomiting at least occasionally as an eating-compensatory behavior (Chapuis-de-Andrade et al., 2017).

A number of genetic, physiological, behavioral, and environmental factors may contribute to the development of eating-compensatory behaviors (American Psychiatric Association, 2013). Especially, ideals of beauty imposed by society, such as having a slim body. Furthermore, demographic characteristics can be associated with engaging in eating-compensatory behaviors, such as race, religion and education. Some data suggested that on average European-Americans tend to be very concerned with thinness and associate it more with beauty than certain ethnic minority groups (Warren et al., 2005). Moreover, Kessler et al. (2013) showed that bulimia nervosa and binge eating disorder are inversely related to educational level when comparing data from several countries. Regarding religion, a study from Northeastern Brazil found association between no religion among female adolescents and an increased risk of developing eating disorders (Vale et al., 2011). Also, the culture can be an important risk factor and must be considered. Authors suggest that the association between beauty and well-being is strongly marked in many societies, especially in the Western world (Rodgers et al., 2011). In Brazil, this is even more evident. For Brazilians, thinness is associated with self-control, bodily perfection and social status of success (Edmonds, 2009).

Additionally, the temperament, when associated with the development of eating disorders, demonstrated to be an important parameter for determining etiology and maintenance of eating-compensatory behaviors (Díaz-Marsá et al., 2000; Martin et al., 2000; Atiye et al., 2015).

Temperament is defined as an emotional expression and behavioral style, which can be observed from early childhood (McAdams and Olson, 2010). In addition, it is characterized as an element with neurochemical correlations (Lara and Akiskal, 2006) and it has strong genetic influences (Gonda et al., 2006; McAdams and Olson, 2010).

To assess the temperament traits Cloninger et al. (1998) and Akiskal et al. (1989) proposed two models that can be conceived as emotional and affective temperaments. The Affective and Emotional Composite Temperament model (AFECT) is a bidimensional model that integrated emotional and affective temperament constructs. In this way, this model is useful in order to better understand affective temperaments in clinical practice (Lara et al., 2006, 2012a). The results of this model show that about 99% of people identify themselves with at least one of the proposed affective temperaments (Lara et al., 2012b). Twelve types of affective temperaments are classified in four groups: (1) externalized types—irritable, disinhibited, and euphoric; (2) internalized types—depressive, avoidant, and apathetic; (3) unstable types—cyclothymic, dysphoric, and volatile; (4) stable types—obsessive, euthymic, and hyperthymic. Although these temperaments are not mutually exclusive since people can have different levels of all 12 temperaments.

Regarding temperament traits and eating-compensatory behaviors, studies show that temperament should be considered as a strategy to build capacity for prevention and treatment of eating disorders (Wagner et al., 2006; Chen et al., 2008; Rodríguez-Cano et al., 2014). For example, people who have bulimia nervosa tend to achieve high scores in impulsiveness and extravagance (Rodríguez-Cano et al., 2014). On the other hand, depressive temperament is strongly associated with eating disorders in general (Rodríguez-Cano et al., 2014).

Indeed, traits of temperaments may be protective or risk factors for the development of psychiatric disorders, depending on the context. Obsessive temperament, for example, is adaptive to environments that require structure and organization, although is more likely to be dysfunctional in situations that require flexibility and innovation (Lara et al., 2012a). Furthermore, affective temperaments are important predictors both of suicide risk and psychopathology and may be used in clinical practice to improve patient care (Pompili et al., 2012).

Thus, it is essential to know about temperament to better understand eating-compensatory behaviors since temperament is related to traits and not only to temporary states or symptoms (Claes et al., 2006; Zwaan et al., 2015). Consequently, it is possible to implement effective strategies for the prevention and treatment of eating disorders.

Considering that Brazilians are a high-risk population for eating-compensatory behaviors and there are few researches in general population, the aim of this study was to evaluate the association between affective temperaments and eating-compensatory behaviors in a large web-based Brazilian sample. Moreover, to the authors' best knowledge, there are no studies in Brazilian sample examining the association between temperament and eating-compensatory behaviors. The principal hypothesis was that cyclothymic, euphoric and volatile types are strongly associated with eating-compensatory behaviors. Our hypothesis is based in authors that found associations between eating disorders and impulsivity and emotion dysregulation (Lian et al., 2017).

Data were collected from an anonymous and voluntary web survey on many psychological and psychiatric measures (Lara et al., 2012b). This type of survey is very appropriate for increasing reports on sensitive and morally charged issues (Turner et al., 1998), such as eating-compensatory behaviors.

## Methods

### Study Design

This is a community cross-sectional study.

### Sample

The sample consisted of 27,501 participants aged between 18 and 55 years old (mean age 28.9 ± 8.7 years, 69.6% women). Most participants were Caucasian (69.5%), they had at least high school degree (95.1%), and they had normal body mass index (45.7% in males and 56.4% in females). Regarding previous diagnosis of anorexia or bulimia nervosa, men reported 0.3% (*n* = 25) and 0.2% (*n* = 17), compared to 0.9% (*n* = 169) and 1.1% (*n* = 207) in women, respectively.

### Instruments

Volunteers answered about eating-compensatory behaviors (fasting, physical exercise, vomiting, medication intake, diuretics and laxatives) with questions such as the following: “Have you ever done exhaustive physical exercise for losing weight? Have you ever done prolonged fasting? Have you ever taken diet pills, laxatives or diuretics? Have you ever induced vomiting?” Answers were graded as follow: never, sometimes (up to twice a month) or frequently (more than 4 times a week). Participants also answered questions such as: “Do you exercise every day? How many times a week do you exercise? In general, how long does each exercise session last? Do you do prolonged fasting every day? How many times a week do you do prolonged fasting? In general, how long does each prolonged fasting that you do? Do you take diet pills, laxatives or diuretics every day for losing weight? How many times a week do you take diet pills, laxatives or diuretics? Do you induce vomiting every day? How many times a week do you induce vomiting?” Thus, the diagnosis of eating-compensatory behaviors was based on the self-report measures of participants.

All volunteers provided basic socio-demographic data (gender, age, race, educational level, religious affiliation, height, current weight, history of bariatric surgery, and previous diagnosis of bulimia and anorexia nervosa).

The affective temperaments were assessed by the AFECT Scale. It is a short, valid, user-friendly and publicly available self-report instrument. Also, it has clear definitions of mental health and dysfunctions that can be easily applied in clinical practice. In this model, both the emotional and affective temperament constructs are integrated. Emotional temperament is understood in six dimensions: volition, anger, inhibition, sensitivity, coping and control, each one divided in two facets. Affective temperaments have 12 constructs.

In total, the AFECTS scale has 62 items and typically takes around 30 min to be completed.

The quality of data was controlled with many validity questions throughout the study. The question “Do you commit to answering the questions honestly?” at the beginning of the questionnaire was used to ensure the participant's sincerity (Mazar and Ariely, 2006). Furthermore, questions focused on the attention with some instruction to be followed, as for example “Please mark the option ‘never' in this question” were distributed throughout the questionnaire. Also, there were the repetition of some questions at different times of the questionnaire. After validity checks, 10.9% of the initial sample were excluded.

### Data Collection

This study and data were based on the Brazilian Internet Study on Temperament and Psychopathology (BRAINSTEP) project (Lara et al., 2012a). This is a web-based anonymous and confidential survey created to investigate temperament, psychiatric disorders, and psychobiological measures in a non-commercial, advertisement-free website (http://www.temperamento.com.br). Internet resource improves and facilitates data collection: all questions have to be answered, no data is missing, and there is no data transfer, which minimizes the possibility of errors. Moreover, large samples are more easily accessed. Several studies were conducted and published using data from BRAINSTEP project.

To participate in the study, the person accesses the project website voluntarily and signs up with an e-mail address. Name and other identification information were not required. Volunteers gave their electronic informed consent for access and permission to use the data before accessing the questionnaires. Individuals could cancel their participation at any moment without justification. The participants had no contact with the researchers. In return for completing the study, volunteers received feedback on their temperament profile at the email address registered at the beginning of the study. Participants answered the instruments from November 2010 to December 2015.

Demographic data was the first scale of this system, after followed by the AFECTS scale.

The Institutional Review Board of São Lucas Hospital reviewed and approved the protocol used for this study (number 1 383 399). All the procedures were adopted to satisfy the National Research Council of Brazil (Resolution 466/2012) and the Code of Ethics of the World Association.

### Data Analysis

The proportion of eating-compensatory behaviors between groups and the relationship between the 12 affective temperaments were analyzed with the Chi-square test. Assumptions for this analysis, such as cells as frequencies, mutually exclusive, independency between groups, among others were taken into account. In this way, one hypothesis regards temperament dependency/independency. If individuals could meet for multiple temperamental styles, some bias can occur. Therefore, multiple regression analysis examined the relationships between the variables. Thus, Wald χ^2^ was employed to examine the role of each variable in a multinomial logistic regression (in the prediction of eating-compensatory behaviors through temperaments as dimensional predictors in the analysis). Analyses were conducted using Statistical Package for Social Sciences, SPSS (Version 22, IBM Corp, Armonk, NY, USA). Risk ratios with 95% confidence intervals (95%CI) for having eating-compensatory behaviors were calculated using multinomial logistic regression analysis. These analyses were adjusted for gender, body mass index, educational level, race, and history of bariatric surgery. Statistical significance was set at *P* < 0.05. In some cases, Bonferroni's correction was applied in most cases to try to compensate the increase by testing each individual hypothesis at a significance level of α.

## Results

The frequency of eating-compensatory behaviors groups for men and women was significantly different in all groups (*p* < 0.001, *df* = 1 for all, χ^2^ = 61.18–414.75). The most prevalent eating-compensatory behaviors for men and women were exhaustive physical exercise and prolonged fasting, respectively. Exhaustive physical exercise was the only behavior more often adopted by men. Women do all other behaviors 3–8 times more when compared to men.

On the other hand, age differences were examined. As expected, men (M_ean_ = 27, SD = 10.17) were older than women (M_ean_ = 26.66, SD = 10.17), which reached statistically significance (*p* < 0.001). Both bulimia and anorexia nervosa groups were evaluated in terms of age through independent samples *t*-test: control (M_ean_ = 26.78, SD = 10.19) vs. bulimia (M_ean_ = 24.45, SD = 8.69), and this reached statistically significance (*p* < 0.001); control (M_ean_ = 26.78, SD = 10.19) vs. anorexia nervosa (M_ean_ = 25.42, SD = 9.68), and this also reached statistically significance (*p* < 0.005). If these differences were examined across sex in bulimia, men were older (M_ean_ = 26.16, SD = 8.05) than women (M_ean_ = 24.35, SD = 8.70). However, only women reached statistical significance vs. their control group (*p* < 0.001). Although, the inverse pattern was found for differences across sex in anorexia nervosa, men were younger (M_ean_ = 24.26, SD = 7.63) than women (M_ean_ = 25.56, SD = 9.90), and both groups reached statistical significance vs. their control group (*p* < 0.001).

Tables 1, 2 show that religion had no statistical significance for men and little effect sizes for women. Among men, lower educational level was associated with less practice of exhaustive exercise and medication intake. However, they presented a higher risk to adopt vomiting to lose weight. For women, having a low educational attainment was associated with a higher risk of prolonged fasting and vomiting, but a lower-risk adopting all other behaviors analyzed.

**Table 1:** Demographic data and risk ratios for disordered eating behaviors for men.

**Variable**	**Total population**	**Prolonged fasting**			**Exhaustive exercise**			**Laxatives**			**Diuretics**			**Medication**			**Vomiting**		
	**%**	**% ^a^**	**OR**	**95% CI**	**% ^a^**	**OR**	**95% CI**	**% ^a^**	**OR**	**95% CI**	**OR**	**% ^a^**	**95% CI**	**% ^a^**	**OR**	**95% CI**	**% ^a^**	**OR**	**95% CI**
**Race**																			
White	69.5	3.9	1	NA	10.6	1	NA	0.8	1		1.0	1		2.1	1		0.5	1	
Mixed	22.8	3.5	0.87	(0.66–1.15)	8.4	0.87	(0.66–1.15)	1.0	1.26	(0.74–2.16)	0.8	0.86	(0.49–1.49)	1.9	0.87	(0.60–1.28)	0.8	1.37	(0.73–2.58)
Afro-Brazilian	4.9	3.6	0.91	(0.53–1.55)	9.7	0.91	(0.53–1.55)	0.7	0.92	(0.29–2.98)	1.0	1.02	(0.37–2.82)	2.2	1.00	(0.50–2.01)	0.2	0.44	(0.6–3.24)
Asian	0.9	6.8	2.09	(0.82–5.31)	15.1	2.09	(0.82–5.31)	1.4	1.76	(0.24–12.93)	1.4	1.54	(0.21–11.26)	0.0	0		1.4	2.47	(0.33–18.62)
Other	1.9	2.5	0.60	(0.22–1.64)	11.8	0.60	(0.22–1.64)	0.6	0.79	(0.11–5.77)	1.2	1.30	(0.32–5.39)	0.6	0.32	(0.04–2.31)	0.6	1.20	(0.16–8.91)
**Educational level**																			
Elementary school	5.7	5.4	1.45	(0.93–2.25)	7.9	0.59^*^	(0.41–0.84)	1.3	1.31	(0.54–3.19)	0.8	0.86	(0.30–2.46)	0.6	0.23^*^	(0.07–0.75)	1.0	3.16^*^	(1.03–9.70)
High school	58.2	3.8	1.06	(0.83–1.35)	9.8	0.82^*^	(0.70–0.96)	0.7	0.79	(0.48–1.30)	1.0	1.03	(0.64–1.64)	2.0	0.83	(0.59–1.17)	0.7	2.09	(0.96–4.58)
College degree	36.1	3.6	1		10.8	1		0.9	1		0.9	1		2.3	1		0.3	1	
**Religion**																			
Catholic	30.4	3.3	1		10.2	1		0.8	1		0.7	1		1.9	1		0.2	1	
Evangelical	17.5	3.4	1.10	(0.77- 1.58)	11.2	1.08	(0.87–1.34)	1.1	1.34	(0.70–2.57)	1.2	1.66	(0.87–3.18)	2.6	1.47	(0.95–2.28)	0.6	2.67	(0.88–8.05)
Spiritist	8.5	3.4	1.08	(0.68- 1.72)	11.1	1.04	(0.79–1.37)	1.0	1.22	(0.52–2.89)	1.6	2.12^*^	(1.01–4.47)	3.5	1.91^*^	(1.16–3.14)	1.1	5.81^*^	(1.88–17.91)
Jewish	0.3	0.0	0	NA	10.7	1.22	(0.34–4.35)	3.6	4.34	(0.56–33.51)	0.0	0	NA	0.0	0	NA	0.0	0	NA
Other	7.5	4.0	1.22	(0.77- 1.93)	10.0	0.92	(0.68–1.24)	0.5	0.59	(0.17–1.97)	0.8	1.06	(0.40–2.85)	2.7	1.47	(0.89–2.77)	0.8	3.65^*^	(1.05–12.71)
No religion	35.7	4.6	1.42^*^	(1.08–1.88)	9.2	0.82^*^	(0.68–0.99)	0.7	0.88	(0.48–1.61)	0.9	1.14	(0.63–2.07)	1.3	0.70	(0.46–1.08)	0.7	3.14^*^	(1.17–8.40)
Total	100	3.8	NA	NA	3.8	NA	NA	0.8	NA	NA	0.9	NA	NA	2.0	NA	NA	0.6	NA	NA

a *Percentage of corresponding n for total population. NA, not applicable. Odds ratios (OR) are statistically significant (P < 0.05) when 1.0 is not included in the 95% confidence intervals; statistically significant odds ratios are marked with an asterisk*.

**Table 2:** Demographic data and risk ratios for disordered eating behaviors for women.

**Variable**	**Total population**	**Prolonged fasting**			**Exhaustive exercise**			**Laxatives**			**Diuretics**			**Medication**			**Vomiting**		
	**%**	**% ^a^**	**OR**	**95% CI**	**% ^a^**	**OR**	**95% CI**	**% ^a^**	**OR**	**95% CI**	**% ^a^**	**OR**	**95% CI**	**% ^a^**	**OR**	**95% CI**	**% ^a^**	**OR**	**95% CI**
**Race**																			
White	70.2	10.6	1		7.3	1		6.9	1		5.1	1		8.7	1		3.4	1	
Mixed	22.4	10.3	0.95	(0.85–1.07)	7.0	0.95	(0.85–1.07)	5.9	0.83^*^	(0.72–0.96)	4.9	0.91	(0.77–1.07)	7.0	0.79^*^	(0.69–0.91)	3.1	0.89	(0.72–1.08)
Afro-Brazilian	4.7	9.8	0.94	(0.74–1.18)	8.0	0.94	(0.74–1.18)	5.9	0.87	(0.65–1.15)	5.0	0.96	(0.71–1.32)	8.5	0.98	(0.77–1.26)	3.4	0.99	(0.68–1.44)
Asian	1.1	11.9	1.17	(0.76–1.80)	11.4	1.17	(0.76–1.80)	5.0	0.67	(0.36–1.24)	5.5	1.06	(0.77–1.07)	6.4	0.70	(0.40–1.22)	5.0	1.44	(0.77–2.67)
Other	1.6	6.7	0.61^*^	(0.38–0.97)	6.7	0.61^*^	(0.38–0.97)	4.7	0.70	(0.40–1.20)	2.0	0.37^*^	(0.17–0.84)	4.0	0.43^*^	(0.24–0.77)	3.7	1.04	(0.56–1.92)
**Educational level**																			
Elementary school	4.6	11.7	1.39^*^	(1.11–1.74)	4.5	0.54^*^	(0.39–0.76)	6.1	0.88	(0.66–1.19)	5.4	0.98	(0.72–1.34)	8.5	0.84	(0.65–1.10)	3.9	1.41	(0.97–2.06)
High school	52.5	11.5	1.32^*^	(1.20–1.46)	7.5	1.01	(0.90–1.13)	6.4	0.88^*^	(0.79–0.99)	4.6	0.80^*^	(0.70–0.92)	7.1	0.86^*^	(0.77–0.97)	3.7	1.28^*^	(1.07–1.54)
College degree	43	9.1	1		7.3	1		6.8	1		5.4	1		9.6	1		2.8	1	
**Religion**																			
Catholic	32.9	9.0	1		6.7	1		6.4	1		4.4	1		7.6	1		2.5	1	
Evangelical	20.2	10.7	1.28^*^	(1.12–1.47)	6.8	1.03	(0.88–1.22)	6.1	0.97	(0.82–1.15)	5.6	1.29^*^	(1.07–1.55)	8.6	1.15	(0.99–1.34)	2.9	1.19	(0.93–1.53)
Spiritist	13.7	9.3	1.11	(0.94–1.30)	8.0	1.22	(1.02–1.45)	6.9	1.15	(0.96–1.38)	5.0	1.22	(0.99–1.52)	10.5	1.43^*^	(1.22–1.69)	3.4	1.38^*^	(1.06–1.81)
Jewish	0.2	7.0	0.82	(0.25–2.74)	14.0	2.59^*^	(1.04–6.46)	9.3	1.63	(0.57–4.65)	7.0	1.54	(0.47–5.05)	16.3	1.94	(0.82–4.57)	0.0	0	NA
Other	7.1	11.8	1.45^*^	(1.20–1.75)	9.2	1.40	(1.13–1.74)	6.1	1.03	(0.81–1.32)	6.2	1.51^*^	(1.18–1.95)	8.9	1.20	(0.97–1.49)	3.9	1.59^*^	(1.15–2.18)
No religion	25.9	12.3	1.56^*^	(1.38–177)	7.4	1.11	(0.96–1.29)	7.1	1.14	(0.98–1.32)	4.9	1.13	(0.95–1.35)	7.1	1.04	(0.90–1.21)	4.6	1.92^*^	(1.56–2.37)
Total	100	10.5	NA	NA	10.5	NA	NA	6.6	NA	NA	5.0	NA	NA	8.2	NA	NA	3.3	NA	NA

a *Percentage of corresponding n for total population. NA, not applicable, Odds ratios (OR) are statistically significant (P < 0.05) when 1.0 is not included in the 95% confidence intervals; statistically significant odds ratios are marked with an asterisk*.

**Figure 1:**
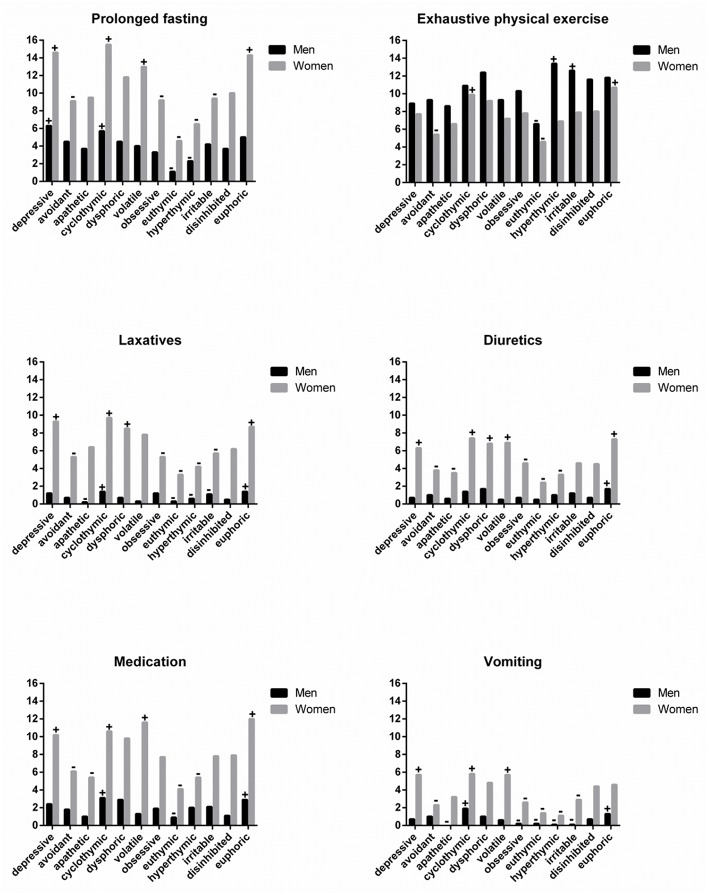
Affective temperaments in disordered eating behaviors groups. Data shown as a percentage (the y axis) of individuals in each category, separated by gender. (+) represents a higher proportion and (–) indicates a lower proportion within each affective temperament type, according to chi-square test (*p* < 0.001).

Figure 1 shows individuals' eating-compensatory behaviors according to their affective temperaments, with significant differences [$\chi_{\left(55\right)}^{2}$ = 189,281 in males, $\chi_{\left(55\right)}^{2}$ = 373,342 in females, *p* < 0.001]. If a Bonferroni's correction would be applied, these results would also reach statistical significance (*p* < 0.008, *m* = 6). Interestingly, cyclothymic women do more all eating-compensatory behaviors analyzed than other emotional types. In addition, depressive women presented this behavior, excepted for exhaustive physical exercise. Among men, cyclothymic types also reported to adopt more frequently eating-compensatory behaviors compared to other affective temperaments.

Although men, in general, do more exhaustive physical exercise for losing weight, hyperthymic and irritable men reported to do it more frequently. The opposite profile was observed in euthymic men. Among women, only cyclothymic and euphoric types engaged in exhaustive physical exercise more frequently than average.

Furthermore, most depressive, cyclothymic and euphoric women (~16%) reported prolonged fasting. Nevertheless, only depressive and cyclothymic men adopted this eating-compensatory behavior more frequently. Laxatives and diuretics intake had a similar standard in women, with depressive, cyclothymic, dysphoric, and euphoric types adopting these behaviors regularly. However, only euphoric men reported diuretic intake to lose weight more frequently. Furthermore, euphoric and cyclothymic men adopted the use of medication and self-induced vomiting more than other types. Among women, depressive, cyclothymic, and volatile women adopted medication intake and vomiting to lose weight.

Odds Ratios (OR) were employed to examine the association between variables. Also, risk ratios (RR) were employed to measure the association between the exposure and the outcome In this way, values of RR equal to 1 means not affection, RR lower than 1 means the risk is decreased, and RR higher than 1 means that the risk is increased. Moreover, confidence intervals are depicted in Table 3 to examine the statistical significance as mentioned before. The highest risk effects were observed for prolonged fasting, both for men and women, but higher in men. Compared to euthymic individuals, internalized types, especially in men, presented higher effect sizes. Laxatives, medication intake and vomiting presented statistical significance only for women (*p* < 0.001), with moderate effect sizes.

**Table 3:** Risk ratios for affective temperaments in frequent disordered eating behaviors.

		**Fasting**	**Physical exercise**	**Laxatives**	**Diuretics**	**Medication**	**Vomiting**
Depressive	Men	7.61 (3.36–17.25)^*^	0.99 (0.67–1.45)	2.08 (0.50–8.61)	0.63 (0.15–2.62)	1.31 (0.54–3.16)	1.37 (0.26–7.35)
	Women	2.34 (1.74–3.13)^*^	0.96 (0.70–1.32)	2.02 (1.38–2.95)^*^	0.86 (0.56–1.33)	1.77 (1.27–2.47)^*^	1.88 (1.15–3.08)^*^
Avoidant	Men	5.73 (2.52–13.05)^*^	1.22 (0.84–1.76)	0.49 (0.09–2.60)	1.20 (0.32 a 4.54)	1.25 (0.52–2.99)	2.42 (0.49–12.07)
	Women	1.60 (1.19–2.16)^*^	0.88 (0.63–1.21)	1.61 (1.09–2.38)^*^	1.02 (0.66–1.59)	1.11 (0.78–1.57)	1.35 (0.80–2.28)
Apathetic	Men	5.08 (2.06–12.56)^*^	1.07 (0.69–1.68)	0.40 (0.40–4.41)	1.56 (0.31–7.80)	0.54 (0.14–2.14)	0.18 (0.06–2.48)
	Women	1.24 (0.83–1.87)	1.00 (0.65–1.54)	2.00 (1.21–3.31)^*^	0.83 (0.45–1.53)	1.01 (0.62–1.64)	1.54 (0.80–2.95)
Cyclothymic	Men	4.18 (1.83–9.53)^*^	1.47 (1.03–2.10)^*^	1.65 (0.41–6.71)	0.94 (0.25–3.44)	1.87 (0.82–4.26)	2.52 (0.53–11.95)
	Women	1.93 (1.47–2.55)^*^	1.55 (1.17–2.07)^*^	1.81 (1.27–2.60)^*^	1.26 (0.85–1.87)	1.96 (1.43–2.68)^*^	1.89 (1.18–3.03)^*^
Cyclothymic^†^	Men	0.89 (0.64–1.25)	0.95 (0.78–1.17)	0.91 (0.44–1.89)	1.20 (0.56–2.60)^*^	0.81 (0.52–1.27)	0.59 (0.28–1.25)
	Women	0.74 (0.67–0.82)^*^	0.76 (0.67–0.86)^*^	0.73 (0.64–0.83)^*^	0.65 (0.56–0.76)^*^	0.80 (0.71–0.90)^*^	0.60 (0.50–0.72)^*^
Dysphoric	Men	3.52 (1.30–9.49)^*^	1.54 (0.95–2.48)	0.52 (0.11–2.72)	3.00 (0.72–12.51)	1.58 (0.54–4.66)	2.55 (0.39–16.48)
	Women	1.19 (0.82–1.73)	1.50 (1.03–2.19)^*^	1.50 (0.92–2.43)	1.16 (0.69–1.95)	2.07 (1.37–3.14)^*^	1.99 (1.12–3.55)^*^
Volatile	Men	5.56 (2.26–13.68)^*^	1.01 (0.63–1.62)	0.45 (0.04–4.88)	0.99 (0.16–6.06)	0.82 (0.26–2.62)	0.58 (0.05–6.73)
	Women	2.12 (1.50–3.01)^*^	1.07 (0.73–1.57)	1.46 (0.92–2.32)	1.12 (0.68–1.87)	1.79 (1.20–2.65)^*^	1.59 (0.90–2.82)
Euthymic	Ref.	1	1	1	1	1	1
Obsessive	Men	3.42 (1.47–7.97)^*^	1.37 (0.96–1.97)	2.65 (0.66–10.60)	0.72 (0.18–2.86)	1.73 (0.74–4.06)	0.24 (0.02–2.81)
	Women	1.50 (1.11–2.01)^*^	1.49 (1.11–2.01)^*^	1.28 (0.87–1.89)	1.24 (0.82–1.89)	1.59 (1.14–2.21)^*^	1.08 (0.64–1.82)
Hyperthymic	Men	2.31 (0.95–5.61)	2.30 (1.60–3.29)^*^	1.15 (0.23–5.61)	1.15 (0.23–5.61)	1.36 (056–3.23)	0.40 (0.03–4.58)
	Women	1.12 (0.78–1.60)	1.63 (1.15–2.31)^*^	1.49 (0.95–2.35)	0.89 (0.52–1.51)	1.04 (0.69–1.57)	0.52 (0.24–1.11)
Irritable	Men	3.93 (1.61–9.60)^*^	1.94 (1.30–2.90)^*^	1.89 (0.38–9.28)	1.39 (0.34–5.72)	1.07 (0.40–2.88)	0.40 (0.03–4.76)
	Women	1.59 (1.15–2.19)^*^	1.58 (1.14–2.19)^*^	1.31 (0.85–2.00)	1.07 (0.67–1.72)	1.47 (1.02–2.12)^*^	1.42 (0.82–2.45)
Desinhibited	Men	4.17 (1.65–10.55)^*^	1.46 (0.93–2.28)	0.63 (0.08–4.79)	1.66 (0.35–7.96)	0.65 (0.18–2.32)	2.55 (0.43–15.23)
	Women	1.30 (0.91–1.86)	1.35 (0.94–1.94)	1.36 (0.85–2.16)	0.91 (0.54–1.54)	1.84 (1.23–2.76)^*^	2.39 (1.38–4.13)^*^
Euphoric	Men	3.64 (1.59–8.76)^*^	1.62 (1.11–2.35)^*^	1.37 (0.32–5.81)	1.73 (0.49–6.13)	1.40 (0.59–3.31)	1.71 (0.33–8.95)
	Women	1.77 (1.30–2.41)^*^	1.84 (1.34–2.51)^*^	1.58 (1.06–2.35)^*^	1.25 (0.81–1.93)	2.02 (1.42–2.85)^*^	1.58 (0.95–2.62)

*Odds ratios are statistically significant (P < 0.05) when 1.0 is not included in the 95% confidence intervals; statistically significant odds ratios are marked with an asterisk*.

† *Cyclothymic temperament excluding people with bipolar disorder (n = 3,581, 874 men, and 2,707 women)*.

Considering that the presence of bipolar disorder might have largely affected the positive response of cyclothymic temperament, a new variable was created. In this way, 127 (12,7%) cyclothymic men and 547 (16,8%) cyclothymic women reported bipolar disorder. These individuals were excluded from this new group and all analyses were reconducted (*n* = 3,581, 874 men, and 2,707 women). When this was accounted for, cyclothymic temperament in disordered eating behaviors reached statistically significance in men just for diuretics intake (Table S1). However, for women, all disordered eating behaviors groups reached statistically significance.

Finally, temperaments were considered as dimensional predictors in the analysis through a multiple regression analysis. Appendix A depicts Wald χ^2^ test and its inherent *p*-values. A total of six models were evaluated on eating-compensatory behaviors. Nagelkerke's pseudo R 2 on the prediction of fasting (0.098), physical exercise (0.030), laxatives (0.095), diuretics (0.072), medication (0.076), and vomiting (0.123). It is remarkable that sex was statistical significant for all models.

**Figure 2:**
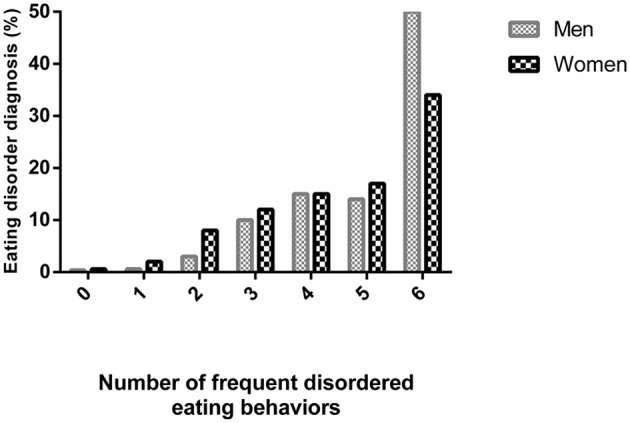
Lifetime diagnosis of eating disorders according to number of frequent disordered eating behaviors.

As shown in Figure 2, the prevalence of a lifetime diagnosis of eating disorders increased steadily from 0 to 5 frequent eating-compensatory behaviors up to ~15% both in men (*p* < 0.001, χ^2^ = 514.029) and women (*p* < 0.001, χ^2^ = 909.170). If a Bonferroni's correction would be applied, these results would also reach statistical significance (*p* < 0.008, *m* = 6). Furthermore, this prevalence doubled for women and tripled for men who adopted all six behaviors. Participants who adopted at least one eating-compensatory behavior frequently represented 0.5 and 0.4% of men, 1.2 and 1.6% of women who received anorexia and bulimia nervosa diagnosis, respectively. For those who adopted six frequently eating-compensatory behaviors, 66.7 and 33.3% of men (*n* = 2 and 1, respectively), 2.6 and 21.1% of women received anorexia and bulimia nervosa diagnosis, respectively.

## Discussion

This study found a complex relationship between eating-compensatory behaviors and affective temperaments. The data showed that the unstable temperament cyclothymic presented higher percentages of eating-compensatory behaviors, for both genders. Even excluding participants who reported having ever received a diagnosis of bipolar disorder—considering that it might have largely affected the positive response of cyclothymic temperament—all disordered eating behaviors groups reached statistically significance for cyclothymic women; although just diuretics intake presented statistical significance for men. In contrast to that, internalized type avoidant and externalized type irritable, in general, adopted lower percentages of eating-compensatory behaviors in women and men, respectively. However, depressive individuals presented a higher risk to adopt prolonged fasting than euthymic individuals did. Consistent with previous studies, the data showed that women adopt more frequently eating-compensatory behaviors (Hoek, 2006; Wells et al., 2006; Hudson et al., 2007; Keski-Rahkonen et al., 2007; Preti et al., 2009). However, these results showed an expressive number of men adopting frequently some eating-compensatory behavior, especially exhaustive physical exercise ~18% (*n* = 1,502). Regarding lifetime diagnosis of eating disorders, even individuals adopting five frequent eating-compensatory behaviors, ~82% of them did not receive a diagnosis of eating disorder, such as anorexia or bulimia nervosa.

Many researchers have studied the association between personality traits and obesity (Sullivan et al., 2007; Martínez et al., 2013; Borelli and Lara, 2014; Leombruni et al., 2014). However, little is known about eating-compensatory behaviors and temperament. Overall, this study suggests that the temperament profile of individuals who adopt frequently eating-compensatory behaviors is very different from those without them. Euphoric and depressive types reported frequently eating-compensatory behaviors more than other temperaments for men and women, respectively. Moreover, cyclothymic types also reported frequently eating-compensatory behaviors, for both genders, what is in line with previous studies (Díaz-Marsá et al., 2000; Zwaan et al., 2015). It is interesting that the opposite profile showed a higher percentage of eating-compensatory behaviors between men and women. Euphoric people are more expansive and impulsive. Depressive people are melancholic and quiet. This difference can be explained by the following: in this study it was investigated if individuals adopt eating-compensatory behaviors and how frequently they do it. Besides that, people more intense, such as low self-esteem individuals can adopt eating-compensatory behaviors and the reasons may be different. Prolonged fasting, for example, is a behavior that can be adopted for a very depressive individual, as well as a euphoric individual can stay for hours without feeding and doing other activities. Regarding cyclothymic types adopt more eating-compensatory behaviors than other temperaments, it can be explained by the ups and downs that individuals face on different occasions. Thus, even if a cyclothymic individual is satisfied with his body weight, he can also show disproportionate and fast reactions as, for example, eating-compensatory behaviors.

On the other hand, irritable and avoidant types presented less prevalence of frequent eating-compensatory behaviors in men and women, respectively. Although the difference between those temperaments, irritable is rated as an externalized type and avoidant is internalized, they present some common characteristics, as suspicious and insecure, what can contribute to the similar pattern showed. Furthermore, to be determined and careful (characteristics of those temperaments) can contribute to not adopt frequent eating-compensatory behaviors, or do it less frequently than a cyclothymic individual for example. Contrasting to this, previous researches found association between eating-compensatory behaviors and different temperaments, especially irritable (Garner et al., 1990; Fassino et al., 2001), avoidant (Baños et al., 2014; Schaumberg et al., 2016) and obsessive types (Kleifield et al., 1994). This difference can be explained by the focus of studies which were based mostly on anorexic (Baños et al., 2014) and bulimic patients (Garner et al., 1990), instead of the general population, such as this sample. Moreover, some studies focused in specific population such as obese (Baños et al., 2014) and overweight (Schaumberg et al., 2016), whereas most participants in this study have normal body mass index. Importantly, in our sample the diagnosis of eating-compensatory behaviors was based on the self-report measures of participants. The individuals answered if they performed some eating-compensatory behavior. They also reported on whether or not a given diagnosis had been assigned to them. Considering that eating-compensatory behaviors are a sensitive and frowned upon issue, participants may not have answered sincerely regarding their actions. Although authors highlighted that a web-based anonymous and confidential survey is very appropriate for it (Turner et al., 1998). Regarding demographic data, this study is in line with previous research that found no relationships between religion or race and eating-compensatory behaviors (Reagan and Hersch, 2005). Additionally, authors investigated racial and gender differences in students with eating disorders, where just gender showed robust OR, race was not statistically significant (Cha et al., 2016). However, some studies indicate that white women have a higher risk of eating disorders compared to black women (DeBate et al., 2001; Roberts et al., 2006). Thus, black people are also in risk to adopt eating-compensatory behaviors once body dissatisfaction can be present in them, as highlighted by other authors (Mchiza et al., 2015). Therefore, considering that effect size was not large for race, even in studies that found an association between white women and eating disorders, it is possible that culture has more relationship to eating-compensatory behaviors than race.

Educational level did not show a robust effect size regarding frequent eating-compensatory behaviors, what is in line with prior researches (Mitchison and Hay, 2014; Hay et al., 2015). However, it is important to note that lower educational level was associated with a higher risk of vomiting, in both genders. The data suggest that individuals with low educational attainment may be particularly vulnerable to purging behaviors, such as self-induced vomiting. Considering this is extreme eating-compensatory behavior, it is reasonable to increase information and prevention programs at school about eating disorders consequences, both for physical and mental health. Furthermore, there is evidence that body image is formed in early life and it will influence individuals' behaviors in the future (Benjamin, 1974). Thus, the sooner children have information about eating disorders, the more effective will be the prevention programs and consequently lower will be the rates of these disorders in the future.

Additionally, this study confirmed previous reports showing that men need to adopt more eating-compensatory behaviors to receive a diagnosis of eating disorders (Räisänen and Hunt, 2014). This finding can be explained by eating disorders usually being associated only to women. Moreover, professionals have more difficulty in diagnosing men since the physiological changes are most evident in women (Støving et al., 2011), such as endocrine manifestations. However, diagnosis prevalence was low even for women in our sample, which is broadly consistent with earlier data (Hudson et al., 2007). It is estimated that less than a quarter of individuals with eating disorders seek evidence-based treatments (Evans et al., 2011; Hart et al., 2011). The individuals do not recognize themselves as having a problem and, when this happens, physicians commonly miss to identify an eating disorder. Participants had to adopt all 6 behaviors frequently to be recognized as having an eating disorder. Delays to recognize an individual with eating disorder can be very harmful, as it is associated with poorer treatment adherence, more comorbidities and higher mortality risk (American Academy of Pediatrics, 2003). Of great importance is the fact that affective temperaments are important predictors of many psychopathologies and may be used in clinical practice to improve patient care (Pompili et al., 2012).

Limitations of the present study relate primarily to the self-reported data of eating-compensatory behaviors. Participants reported on whether or not a given diagnosis had been assigned to them. This form of assessment might be biased by whether or not individuals were seeking treatment for their eating disorder. However, many large-scale epidemiological studies rely on self-reported data (Dahl et al., 2010). The cross-section design does not allow to establish causal relationships, only concludes about associations, what is inherent to this methodology. Data are only from Brazil, a country of western culture and findings may differ from others. Despite these limitations, this study presents many strengths. The results are based on a large national population-based survey from a total of 27.501 participants, including from underweight to obese participants. Compared to other studies on eating-compensatory behaviors, this sample presents a large number of men, contributing to improve knowledge in this area, which is still little known. Besides that, it is the first study to investigate the association of eating-compensatory behaviors and temperament in Brazilians.

## Conclusion

In conclusion, eating-compensatory behaviors were associated with distinctive affective temperaments. Cyclothymic types were more associated with eating-compensatory behaviors in both genders. However, avoidant and irritable types presented lower percentages of eating-compensatory behaviors in women and men, respectively. These data can better subsidize intervention by health professionals, as a strategy to build capacity for prevention, treatment, and care of eating-compensatory behaviors. According to the assumption that participants who adopt frequent eating-compensatory behaviors are more likely to have dysfunctional affective traits, we suggest that a broad understanding of the individuals is essential. Considering that cyclothymic types are more likely to develop eating-compensatory behaviors, it is important to identify this affective type in high-risk population. Moreover, specific actions should be performed, such as a thorough anamnesis, in order to determine the exact emotional state of the individual. This level of specificity allows professionals to direct their actions into the preventive realm. If, for example, the individual presents more traits associated with anxiety, actions that encourage his participation more effectively can be employed (e.g., group activities). On the other hand, if the individual presents a more introspective behavior, actions focused on exploring his own thoughts can be deemed more appropriated. Further studies should focus on eating-compensatory behaviors and affective temperaments to confirm and complement the findings.

## Ethics Statement

The Institutional Review Board of São Lucas Hospital reviewed and approved the protocol used for this study (number 1 383 399). All subjects gave written informed consent in accordance with the Declaration of Helsinki. All the procedures were adopted to satisfy the National Research Council of Brazil (Resolution 466/2012) and the Code of Ethics of the World Association.

## Author Contributions

SC-d-A and DL conceived of the presented idea, developed the theory, performed the computations as well as the analytic calculations and took the lead in writing the manuscript. CM-T verified the analytical methods and participated in drafting the manuscript. DC, FA, and TI encouraged and supervised the findings of this work. All authors discussed the results and contributed to the final manuscript.

### Conflict of Interest Statement

The authors declare that the research was conducted in the absence of any commercial or financial relationships that could be construed as a potential conflict of interest.
