# Supplementary material for: The Association Between Eating-Compensatory Behaviors and Affective Temperament in a Brazilian Population
Source: Front Psychol. 2019 Aug 23;10:1924. doi: 10.3389/fpsyg.2019.01924 (PMC6736602; doi:10.3389/fpsyg.2019.01924)
Supplement: Supplementary file 1 [file Table_1.docx]

**Supplementary material**

**Table S1**

Affective temperaments in disordered eating behaviors groups.

|  |  | Fasting (%) | Physical Exercise (%) | Laxatives (%) | Diuretics (%) | Medication (%) | Vomiting (%) |
| --- | --- | --- | --- | --- | --- | --- | --- |
| Depressive | Men | 6.3* | 8.9 | 1.2 | 0.7 | 2.4 | 0.7 |
|  | Women | 14.6* | 7.7 | 9.3* | 6.3* | 10.2* | 5.7* |
| Avoidant | Men | 4.5 | 9.3 | 0.7 | 1.0 | 1.8 | 1.0 |
|  | Women | 9.1^†^ | 5.4^†^ | 5.3^†^ | 3.8^†^ | 6.1^†^ | 2.3^†^ |
| Apathetic | Men | 3.7 | 8.6 | 0.2^†^ | 0.6 | 1.0 | 0.0^†^ |
|  | Women | 9.5 | 6.6 | 6.4 | 3.5^†^ | 5.4^†^ | 3.2 |
| Cyclothymic | Men | 5.7* | 10.9 | 1.4* | 1.4 | 3.1* | 1.9* |
|  | Women | 15.5* | 9.9* | 9.7* | 7.4* | 10.6* | 5.8* |
| Cyclothymic^‡^ | Men | 3.8 | 10.1 | 0.8 | 0.9* | 2.0 | 0.6 |
|  | Women | 10.5* | 7.3 | 6.6 | 5.0* | 8.2 | 3.3* |
| Dysphoric | Men | 4.5 | 12.4 | 0.7 | 1.7 | 2.9 | 1.0 |
|  | Women | 11.8 | 9.2 | 8.5* | 6.8* | 9.8 | 4.8 |
| Volatile | Men | 4.0 | 9.3 | 0.3 | 0.5 | 1.3 | 0.6 |
|  | Women | 13.0* | 7.2 | 7.8 | 6.9* | 11.6* | 5.7* |
| Obsessive | Men | 3.3 | 10.3 | 1.2 | 0.7 | 1.9 | 0.2^†^ |
|  | Women | 9.2^†^ | 7.8 | 5.3^†^ | 4.6^†^ | 7.7 | 2.6^†^ |
| Euthymic | Men | 1.1^†^ | 6.6^†^ | 0.3^†^ | 0.5 | 0.9^†^ | 0.2^†^ |
|  | Women | 4.6^†^ | 4.6^†^ | 3.3^†^ | 2.4^†^ | 4.1^†^ | 1.4^†^ |
| Hyperthymic | Men | 2.3^†^ | 13.4* | 0.6^†^ | 1.0 | 2.0 | 0.1^†^ |
|  | Women | 6.5^†^ | 6.9 | 4.2^†^ | 3.3^†^ | 5.4^†^ | 1.1^†^ |
| Irritable | Men | 4.2 | 12.6* | 1.1^†^ | 1.2 | 2.1 | 0.1^†^ |
|  | Women | 9.4^†^ | 7.9 | 5.7^†^ | 4.6 | 7.8 | 2.9^†^ |
| Desinhibited | Men | 3.7 | 11.6 | 0.5 | 0.7 | 1.1 | 0.7 |
|  | Women | 10.0 | 8.0 | 6.2 | 4.5 | 7.9 | 4.4 |
| Euphoric | Men | 5.0 | 11.8 | 1.4* | 1.7* | 2.9* | 1.3* |
|  | Women | 14.3* | 10.7* | 8.7* | 7.3* | 12.0* | 4.6 |

* represents a higher proportion within each affective temperament type, according to chi-square test (p<0.001).

^†^ indicates a lower proportion within each affective temperament type, according to chi-square test (p<0.001).

^‡^ Cyclothymic temperament excluding people with bipolar disorder (n=3581, 874 men and 2707 women).
