# Supplementary material for: The Association Between Eating-Compensatory Behaviors and Affective Temperament in a Brazilian Population
Source: Front Psychol. 2019 Aug 23;10:1924. doi: 10.3389/fpsyg.2019.01924 (PMC6736602; doi:10.3389/fpsyg.2019.01924)
Supplement: Supplementary file 2 [file Data_Sheet_1.docx]

**Appendix A**

| **Table 1**  Fasting Prediction | | | | | | | | | |
| --- | --- | --- | --- | --- | --- | --- | --- | --- | --- |
| Fasting prediction | | B | Standard error | Wald | gl | Sig. | Exp(B) | 95% Confidence interval Exp(B) | |
|  |  |  |  |  |  |  |  | Lower level | Upper level |
| Never | Intercetion | .396 | .102 | 15.133 | 1 | .000 |  |  |  |
|  | [ATdep=1] | .845 | .069 | 151.428 | 1 | .000 | 2.328 | 2.035 | 2.664 |
|  | [ATdep=2] | .683 | .063 | 117.756 | 1 | .000 | 1.979 | 1.749 | 2.239 |
|  | [ATdep=3] | .542 | .055 | 98.151 | 1 | .000 | 1.719 | 1.544 | 1.913 |
|  | [ATdep=4] | .330 | .047 | 48.577 | 1 | .000 | 1.390 | 1.267 | 1.525 |
|  | [ATdep=5] | 0^b^ | . | . | 0 | . | . | . | . |
|  | [ATans=1] | -.185 | .062 | 8.850 | 1 | .003 | .831 | .735 | .939 |
|  | [ATans=2] | -.076 | .057 | 1.777 | 1 | .183 | .927 | .828 | 1.036 |
|  | [ATans=3] | -.128 | .051 | 6.223 | 1 | .013 | .880 | .795 | .973 |
|  | [ATans=4] | -.014 | .047 | .090 | 1 | .764 | .986 | .899 | 1.081 |
|  | [ATans=5] | 0^b^ | . | . | 0 | . | . | . | . |
|  | [ATapat=1] | -.004 | .063 | .003 | 1 | .955 | .996 | .881 | 1.127 |
|  | [ATapat=2] | .058 | .061 | .883 | 1 | .347 | 1.059 | .939 | 1.194 |
|  | [ATapat=3] | .042 | .058 | .525 | 1 | .469 | 1.043 | .931 | 1.169 |
|  | [ATapat=4] | .120 | .054 | 4.850 | 1 | .028 | 1.127 | 1.013 | 1.254 |
|  | [ATapat=5] | 0^b^ | . | . | 0 | . | . | . | . |
|  | [ATobses=1] | .217 | .058 | 14.080 | 1 | .000 | 1.243 | 1.109 | 1.392 |
|  | [ATobses=2] | .222 | .058 | 14.935 | 1 | .000 | 1.249 | 1.116 | 1.398 |
|  | [ATobses=3] | .165 | .053 | 9.821 | 1 | .002 | 1.180 | 1.064 | 1.308 |
|  | [ATobses=4] | .158 | .047 | 11.483 | 1 | .001 | 1.171 | 1.069 | 1.282 |
|  | [ATobses=5] | 0^b^ | . | . | 0 | . | . | . | . |
|  | [ATciclo=1] | .400 | .075 | 28.805 | 1 | .000 | 1.492 | 1.289 | 1.727 |
|  | [ATciclo=2] | .353 | .074 | 23.089 | 1 | .000 | 1.424 | 1.233 | 1.645 |
|  | [ATciclo=3] | .216 | .061 | 12.410 | 1 | .000 | 1.241 | 1.100 | 1.399 |
|  | [ATciclo=4] | .253 | .050 | 25.364 | 1 | .000 | 1.288 | 1.167 | 1.422 |
|  | [ATciclo=5] | 0^b^ | . | . | 0 | . | . | . | . |
|  | [ATdisf=1] | .321 | .076 | 17.580 | 1 | .000 | 1.378 | 1.186 | 1.601 |
|  | [ATdisf=2] | .325 | .073 | 19.698 | 1 | .000 | 1.384 | 1.199 | 1.598 |
|  | [ATdisf=3] | .224 | .060 | 14.073 | 1 | .000 | 1.251 | 1.113 | 1.406 |
|  | [ATdisf=4] | .192 | .047 | 16.823 | 1 | .000 | 1.212 | 1.105 | 1.328 |
|  | [ATdisf=5] | 0^b^ | . | . | 0 | . | . | . | . |
|  | [ATeut=1] | -.568 | .078 | 53.391 | 1 | .000 | .567 | .486 | .660 |
|  | [ATeut=2] | -.256 | .079 | 10.606 | 1 | .001 | .774 | .663 | .903 |
|  | [ATeut=3] | -.243 | .080 | 9.232 | 1 | .002 | .785 | .671 | .917 |
|  | [ATeut=4] | -.090 | .081 | 1.230 | 1 | .267 | .914 | .781 | 1.071 |
|  | [ATeut=5] | 0^b^ | . | . | 0 | . | . | . | . |
|  | [ATirrit=1] | .043 | .070 | .372 | 1 | .542 | 1.044 | .910 | 1.197 |
|  | [ATirrit=2] | .064 | .062 | 1.092 | 1 | .296 | 1.066 | .945 | 1.203 |
|  | [ATirrit=3] | .049 | .050 | .964 | 1 | .326 | 1.051 | .952 | 1.160 |
|  | [ATirrit=4] | .049 | .046 | 1.169 | 1 | .280 | 1.051 | .961 | 1.149 |
|  | [ATirrit=5] | 0^b^ | . | . | 0 | . | . | . | . |
|  | [ATvolat=1] | .342 | .063 | 29.233 | 1 | .000 | 1.408 | 1.244 | 1.594 |
|  | [ATvolat=2] | .257 | .064 | 16.180 | 1 | .000 | 1.293 | 1.141 | 1.465 |
|  | [ATvolat=3] | .216 | .059 | 13.486 | 1 | .000 | 1.241 | 1.106 | 1.393 |
|  | [ATvolat=4] | .133 | .052 | 6.479 | 1 | .011 | 1.142 | 1.031 | 1.265 |
|  | [ATvolat=5] | 0^b^ | . | . | 0 | . | . | . | . |
|  | [ATdesin=1] | .023 | .060 | .141 | 1 | .708 | 1.023 | .909 | 1.151 |
|  | [ATdesin=2] | .019 | .061 | .100 | 1 | .752 | 1.019 | .905 | 1.148 |
|  | [ATdesin=3] | .104 | .057 | 3.351 | 1 | .067 | 1.109 | .993 | 1.240 |
|  | [ATdesin=4] | .034 | .050 | .469 | 1 | .494 | 1.035 | .938 | 1.142 |
|  | [ATdesin=5] | 0^b^ | . | . | 0 | . | . | . | . |
|  | [AThipertim=1] | .129 | .072 | 3.208 | 1 | .073 | 1.138 | .988 | 1.311 |
|  | [AThipertim=2] | .195 | .072 | 7.361 | 1 | .007 | 1.215 | 1.056 | 1.399 |
|  | [AThipertim=3] | .210 | .070 | 8.954 | 1 | .003 | 1.234 | 1.075 | 1.416 |
|  | [AThipertim=4] | .206 | .070 | 8.586 | 1 | .003 | 1.229 | 1.071 | 1.411 |
|  | [AThipertim=5] | 0^b^ | . | . | 0 | . | . | . | . |
|  | [ATCeuf=1] | .474 | .059 | 65.526 | 1 | .000 | 1.606 | 1.432 | 1.801 |
|  | [ATCeuf=2] | .446 | .060 | 54.590 | 1 | .000 | 1.562 | 1.388 | 1.758 |
|  | [ATCeuf=3] | .345 | .056 | 37.948 | 1 | .000 | 1.412 | 1.265 | 1.575 |
|  | [ATCeuf=4] | .207 | .053 | 15.367 | 1 | .000 | 1.230 | 1.109 | 1.364 |
|  | [ATCeuf=5] | 0^b^ | . | . | 0 | . | . | . | . |
|  | [sex=1] | 1.210 | .048 | 633.154 | 1 | .000 | 3.353 | 3.051 | 3.684 |
|  | [sex=2] | 0^b^ | . | . | 0 | . | . | . | . |
| Sometimes | Interception | .154 | .108 | 2.032 | 1 | .154 |  |  |  |
|  | [ATdep=1] | .508 | .073 | 48.450 | 1 | .000 | 1.662 | 1.441 | 1.918 |
|  | [ATdep=2] | .530 | .066 | 63.787 | 1 | .000 | 1.699 | 1.492 | 1.935 |
|  | [ATdep=3] | .368 | .058 | 40.370 | 1 | .000 | 1.445 | 1.290 | 1.619 |
|  | [ATdep=4] | .233 | .050 | 21.516 | 1 | .000 | 1.262 | 1.144 | 1.392 |
|  | [ATdep=5] | 0^b^ | . | . | 0 | . | . | . | . |
|  | [ATans=1] | -.236 | .066 | 12.662 | 1 | .000 | .789 | .693 | .899 |
|  | [ATans=2] | -.079 | .060 | 1.692 | 1 | .193 | .924 | .821 | 1.041 |
|  | [ATans=3] | -.122 | .054 | 5.030 | 1 | .025 | .885 | .795 | .985 |
|  | [ATans=4] | -.016 | .050 | .110 | 1 | .740 | .984 | .892 | 1.084 |
|  | [ATans=5] | 0^b^ | . | . | 0 | . | . | . | . |
|  | [ATapat=1] | .056 | .067 | .697 | 1 | .404 | 1.057 | .928 | 1.205 |
|  | [ATapat=2] | .070 | .065 | 1.169 | 1 | .280 | 1.073 | .944 | 1.218 |
|  | [ATapat=3] | .095 | .062 | 2.375 | 1 | .123 | 1.100 | .974 | 1.241 |
|  | [ATapat=4] | .130 | .058 | 5.057 | 1 | .025 | 1.138 | 1.017 | 1.275 |
|  | [ATapat=5] | 0^b^ | . | . | 0 | . | . | . | . |
|  | [ATobses=1] | .119 | .062 | 3.745 | 1 | .053 | 1.127 | .998 | 1.271 |
|  | [ATobses=2] | .189 | .061 | 9.647 | 1 | .002 | 1.208 | 1.072 | 1.360 |
|  | [ATobses=3] | .116 | .056 | 4.326 | 1 | .038 | 1.123 | 1.007 | 1.253 |
|  | [ATobses=4] | .126 | .049 | 6.569 | 1 | .010 | 1.134 | 1.030 | 1.249 |
|  | [ATobses=5] | 0^b^ | . | . | 0 | . | . | . | . |
|  | [ATciclo=1] | .178 | .079 | 5.032 | 1 | .025 | 1.195 | 1.023 | 1.396 |
|  | [ATciclo=2] | .321 | .077 | 17.231 | 1 | .000 | 1.378 | 1.185 | 1.604 |
|  | [ATciclo=3] | .163 | .065 | 6.326 | 1 | .012 | 1.177 | 1.037 | 1.337 |
|  | [ATciclo=4] | .190 | .053 | 12.757 | 1 | .000 | 1.209 | 1.090 | 1.342 |
|  | [ATciclo=5] | 0^b^ | . | . | 0 | . | . | . | . |
|  | [ATdisf=1] | .169 | .081 | 4.345 | 1 | .037 | 1.185 | 1.010 | 1.389 |
|  | [ATdisf=2] | .218 | .077 | 7.903 | 1 | .005 | 1.243 | 1.068 | 1.447 |
|  | [ATdisf=3] | .137 | .063 | 4.668 | 1 | .031 | 1.147 | 1.013 | 1.298 |
|  | [ATdisf=4] | .191 | .049 | 15.004 | 1 | .000 | 1.211 | 1.099 | 1.334 |
|  | [ATdisf=5] | 0^b^ | . | . | 0 | . | . | . | . |
|  | [ATeut=1] | -.397 | .082 | 23.231 | 1 | .000 | .672 | .572 | .790 |
|  | [ATeut=2] | -.195 | .083 | 5.507 | 1 | .019 | .823 | .699 | .968 |
|  | [ATeut=3] | -.220 | .085 | 6.761 | 1 | .009 | .803 | .680 | .947 |
|  | [ATeut=4] | -.064 | .085 | .561 | 1 | .454 | .938 | .794 | 1.109 |
|  | [ATeut=5] | 0^b^ | . | . | 0 | . | . | . | . |
|  | [ATirrit=1] | .102 | .074 | 1.908 | 1 | .167 | 1.108 | .958 | 1.281 |
|  | [ATirrit=2] | .125 | .065 | 3.692 | 1 | .055 | 1.133 | .997 | 1.287 |
|  | [ATirrit=3] | .061 | .053 | 1.292 | 1 | .256 | 1.063 | .957 | 1.180 |
|  | [ATirrit=4] | .122 | .048 | 6.422 | 1 | .011 | 1.130 | 1.028 | 1.242 |
|  | [ATirrit=5] | 0^b^ | . | . | 0 | . | . | . | . |
|  | [ATvolat=1] | .132 | .067 | 3.863 | 1 | .049 | 1.141 | 1.000 | 1.302 |
|  | [ATvolat=2] | .189 | .067 | 7.833 | 1 | .005 | 1.207 | 1.058 | 1.378 |
|  | [ATvolat=3] | .094 | .063 | 2.244 | 1 | .134 | 1.098 | .972 | 1.241 |
|  | [ATvolat=4] | .111 | .055 | 4.102 | 1 | .043 | 1.118 | 1.004 | 1.245 |
|  | [ATvolat=5] | 0^b^ | . | . | 0 | . | . | . | . |
|  | [ATdesin=1] | -.038 | .064 | .356 | 1 | .551 | .962 | .849 | 1.091 |
|  | [ATdesin=2] | .032 | .064 | .246 | 1 | .620 | 1.032 | .910 | 1.171 |
|  | [ATdesin=3] | .076 | .060 | 1.597 | 1 | .206 | 1.079 | .959 | 1.214 |
|  | [ATdesin=4] | .040 | .053 | .573 | 1 | .449 | 1.041 | .938 | 1.155 |
|  | [ATdesin=5] | 0^b^ | . | . | 0 | . | . | . | . |
|  | [AThipertim=1] | .110 | .077 | 2.046 | 1 | .153 | 1.116 | .960 | 1.297 |
|  | [AThipertim=2] | .207 | .076 | 7.339 | 1 | .007 | 1.230 | 1.059 | 1.428 |
|  | [AThipertim=3] | .184 | .075 | 6.030 | 1 | .014 | 1.201 | 1.038 | 1.391 |
|  | [AThipertim=4] | .253 | .075 | 11.517 | 1 | .001 | 1.288 | 1.113 | 1.490 |
|  | [AThipertim=5] | 0^b^ | . | . | 0 | . | . | . | . |
|  | [ATCeuf=1] | .296 | .062 | 22.680 | 1 | .000 | 1.344 | 1.190 | 1.518 |
|  | [ATCeuf=2] | .301 | .064 | 22.359 | 1 | .000 | 1.352 | 1.193 | 1.532 |
|  | [ATCeuf=3] | .209 | .059 | 12.474 | 1 | .000 | 1.233 | 1.098 | 1.385 |
|  | [ATCeuf=4] | .112 | .056 | 4.028 | 1 | .045 | 1.119 | 1.003 | 1.248 |
|  | [ATCeuf=5] | 0^b^ | . | . | 0 | . | . | . | . |
|  | [sex=1] | .466 | .051 | 82.654 | 1 | .000 | 1.594 | 1.441 | 1.762 |
|  | [sex=2] | 0^b^ | . | . | 0 | . | . | . | . |

ATdep: Affective temperament depressive; ATans: Affective temperament avoidant; ATapat: Affective temperament apathetic; ATobses: Affective temperament obsessive; ATciclo: Affective temperament cyclothymic; ATdisf: Affective temperament dysphoric; ATeut: Affective temperament euthymic; ATirrit: Affective temperament irritable; ATvolat: Affective temperament volatile; ATdesin: Affective temperament disinhibited; AThipertim: Affective temperament hyperthymic; ATCeuf: Affective temperament euphoric; Sex=1: Men; Sex=2: Women.

| **Table 2**  Physical Exercise Prediction | | | | | | | | | |
| --- | --- | --- | --- | --- | --- | --- | --- | --- | --- |
| Physical Exercise | | B | Standard error | Wald | gl | Sig. | Exp(B) | 95% Confidence interval Exp(B) | |
|  |  |  |  |  |  |  |  | Lower level | Upper level |
| Never | Interception | .933 | .099 | 88.576 | 1 | .000 |  |  |  |
|  | [ATdep=1] | .067 | .067 | 1.002 | 1 | .317 | 1.070 | .938 | 1.220 |
|  | [ATdep=2] | .118 | .064 | 3.431 | 1 | .064 | 1.125 | .993 | 1.275 |
|  | [ATdep=3] | .121 | .059 | 4.225 | 1 | .040 | 1.129 | 1.006 | 1.267 |
|  | [ATdep=4] | .115 | .053 | 4.681 | 1 | .031 | 1.121 | 1.011 | 1.244 |
|  | [ATdep=5] | 0^b^ | . | . | 0 | . | . | . | . |
|  | [ATans=1] | -.067 | .063 | 1.120 | 1 | .290 | .935 | .826 | 1.059 |
|  | [ATans=2] | -.035 | .057 | .369 | 1 | .544 | .966 | .863 | 1.081 |
|  | [ATans=3] | .017 | .054 | .103 | 1 | .748 | 1.017 | .916 | 1.130 |
|  | [ATans=4] | .020 | .048 | .170 | 1 | .680 | 1.020 | .928 | 1.121 |
|  | [ATans=5] | 0^b^ | . | . | 0 | . | . | . | . |
|  | [ATapat=1] | -.136 | .066 | 4.202 | 1 | .040 | .873 | .767 | .994 |
|  | [ATapat=2] | -.138 | .065 | 4.557 | 1 | .033 | .871 | .767 | .989 |
|  | [ATapat=3] | -.108 | .063 | 2.932 | 1 | .087 | .898 | .793 | 1.016 |
|  | [ATapat=4] | .054 | .060 | .789 | 1 | .374 | 1.055 | .937 | 1.187 |
|  | [ATapat=5] | 0^b^ | . | . | 0 | . | . | . | . |
|  | [ATobses=1] | .340 | .062 | 30.039 | 1 | .000 | 1.404 | 1.244 | 1.586 |
|  | [ATobses=2] | .170 | .057 | 8.717 | 1 | .003 | 1.185 | 1.059 | 1.326 |
|  | [ATobses=3] | .273 | .055 | 24.720 | 1 | .000 | 1.314 | 1.180 | 1.463 |
|  | [ATobses=4] | .185 | .046 | 15.822 | 1 | .000 | 1.203 | 1.098 | 1.317 |
|  | [ATobses=5] | 0^b^ | . | . | 0 | . | . | . | . |
|  | [ATciclo=1] | .315 | .070 | 20.313 | 1 | .000 | 1.370 | 1.195 | 1.572 |
|  | [ATciclo=2] | .251 | .070 | 13.042 | 1 | .000 | 1.286 | 1.122 | 1.473 |
|  | [ATciclo=3] | .233 | .062 | 13.898 | 1 | .000 | 1.262 | 1.117 | 1.427 |
|  | [ATciclo=4] | .167 | .051 | 10.510 | 1 | .001 | 1.182 | 1.068 | 1.307 |
|  | [ATciclo=5] | 0^b^ | . | . | 0 | . | . | . | . |
|  | [ATdisf=1] | .296 | .073 | 16.378 | 1 | .000 | 1.344 | 1.165 | 1.551 |
|  | [ATdisf=2] | .228 | .070 | 10.512 | 1 | .001 | 1.256 | 1.094 | 1.442 |
|  | [ATdisf=3] | .206 | .061 | 11.457 | 1 | .001 | 1.229 | 1.091 | 1.385 |
|  | [ATdisf=4] | .107 | .048 | 5.001 | 1 | .025 | 1.113 | 1.013 | 1.223 |
|  | [ATdisf=5] | 0^b^ | . | . | 0 | . | . | . | . |
|  | [ATeut=1] | -.163 | .071 | 5.223 | 1 | .022 | .849 | .738 | .977 |
|  | [ATeut=2] | -.020 | .071 | .084 | 1 | .771 | .980 | .853 | 1.125 |
|  | [ATeut=3] | .053 | .072 | .537 | 1 | .464 | 1.054 | .916 | 1.213 |
|  | [ATeut=4] | .028 | .067 | .177 | 1 | .674 | 1.029 | .901 | 1.174 |
|  | [ATeut=5] | 0^b^ | . | . | 0 | . | . | . | . |
|  | [ATirrit=1] | .089 | .072 | 1.506 | 1 | .220 | 1.093 | .948 | 1.259 |
|  | [ATirrit=2] | .063 | .061 | 1.057 | 1 | .304 | 1.065 | .944 | 1.201 |
|  | [ATirrit=3] | .088 | .052 | 2.885 | 1 | .089 | 1.092 | .987 | 1.208 |
|  | [ATirrit=4] | .140 | .047 | 8.875 | 1 | .003 | 1.150 | 1.049 | 1.260 |
|  | [ATirrit=5] | 0^b^ | . | . | 0 | . | . | . | . |
|  | [ATvolat=1] | .245 | .065 | 13.992 | 1 | .000 | 1.277 | 1.123 | 1.452 |
|  | [ATvolat=2] | .150 | .065 | 5.249 | 1 | .022 | 1.161 | 1.022 | 1.320 |
|  | [ATvolat=3] | .140 | .062 | 5.126 | 1 | .024 | 1.151 | 1.019 | 1.299 |
|  | [ATvolat=4] | .102 | .056 | 3.371 | 1 | .066 | 1.108 | .993 | 1.235 |
|  | [ATvolat=5] | 0^b^ | . | . | 0 | . | . | . | . |
|  | [ATdesin=1] | .096 | .062 | 2.374 | 1 | .123 | 1.101 | .974 | 1.244 |
|  | [ATdesin=2] | .083 | .062 | 1.807 | 1 | .179 | 1.087 | .963 | 1.227 |
|  | [ATdesin=3] | .039 | .058 | .453 | 1 | .501 | 1.040 | .928 | 1.164 |
|  | [ATdesin=4] | .071 | .052 | 1.878 | 1 | .171 | 1.073 | .970 | 1.188 |
|  | [ATdesin=5] | 0^b^ | . | . | 0 | . | . | . | . |
|  | [AThipertim=1] | .359 | .070 | 26.106 | 1 | .000 | 1.432 | 1.248 | 1.643 |
|  | [AThipertim=2] | .315 | .068 | 21.317 | 1 | .000 | 1.370 | 1.198 | 1.565 |
|  | [AThipertim=3] | .310 | .065 | 22.533 | 1 | .000 | 1.364 | 1.200 | 1.550 |
|  | [AThipertim=4] | .188 | .062 | 9.210 | 1 | .002 | 1.207 | 1.069 | 1.364 |
|  | [AThipertim=5] | 0^b^ | . | . | 0 | . | . | . | . |
|  | [ATCeuf=1] | .411 | .061 | 44.957 | 1 | .000 | 1.508 | 1.337 | 1.700 |
|  | [ATCeuf=2] | .278 | .061 | 20.964 | 1 | .000 | 1.320 | 1.172 | 1.486 |
|  | [ATCeuf=3] | .236 | .057 | 17.006 | 1 | .000 | 1.266 | 1.132 | 1.417 |
|  | [ATCeuf=4] | .073 | .053 | 1.881 | 1 | .170 | 1.075 | .969 | 1.193 |
|  | [ATCeuf=5] | 0^b^ | . | . | 0 | . | . | . | . |
|  | [sex=1] | -.401 | .037 | 116.865 | 1 | .000 | .669 | .622 | .720 |
|  | [sex=2] | 0^b^ | . | . | 0 | . | . | . | . |
| Sometimes | Interception | .639 | .104 | 37.997 | 1 | .000 |  |  |  |
|  | [ATdep=1] | .141 | .070 | 4.021 | 1 | .045 | 1.152 | 1.003 | 1.322 |
|  | [ATdep=2] | .159 | .067 | 5.673 | 1 | .017 | 1.172 | 1.029 | 1.336 |
|  | [ATdep=3] | .155 | .062 | 6.339 | 1 | .012 | 1.168 | 1.035 | 1.318 |
|  | [ATdep=4] | .114 | .056 | 4.205 | 1 | .040 | 1.121 | 1.005 | 1.251 |
|  | [ATdep=5] | 0^b^ | . | . | 0 | . | . | . | . |
|  | [ATans=1] | -.064 | .067 | .915 | 1 | .339 | .938 | .823 | 1.069 |
|  | [ATans=2] | .003 | .060 | .003 | 1 | .955 | 1.003 | .892 | 1.128 |
|  | [ATans=3] | .044 | .056 | .615 | 1 | .433 | 1.045 | .936 | 1.167 |
|  | [ATans=4] | .042 | .051 | .694 | 1 | .405 | 1.043 | .945 | 1.152 |
|  | [ATans=5] | 0^b^ | . | . | 0 | . | . | . | . |
|  | [ATapat=1] | -.107 | .070 | 2.381 | 1 | .123 | .898 | .784 | 1.029 |
|  | [ATapat=2] | -.049 | .068 | .526 | 1 | .468 | .952 | .833 | 1.087 |
|  | [ATapat=3] | -.034 | .066 | .260 | 1 | .610 | .967 | .849 | 1.101 |
|  | [ATapat=4] | .120 | .063 | 3.602 | 1 | .058 | 1.127 | .996 | 1.276 |
|  | [ATapat=5] | 0^b^ | . | . | 0 | . | . | . | . |
|  | [ATobses=1] | .186 | .065 | 8.087 | 1 | .004 | 1.204 | 1.059 | 1.368 |
|  | [ATobses=2] | .155 | .060 | 6.647 | 1 | .010 | 1.167 | 1.038 | 1.313 |
|  | [ATobses=3] | .271 | .057 | 22.510 | 1 | .000 | 1.312 | 1.173 | 1.467 |
|  | [ATobses=4] | .168 | .048 | 12.085 | 1 | .001 | 1.183 | 1.076 | 1.301 |
|  | [ATobses=5] | 0^b^ | . | . | 0 | . | . | . | . |
|  | [ATciclo=1] | .117 | .073 | 2.544 | 1 | .111 | 1.124 | .974 | 1.297 |
|  | [ATciclo=2] | .186 | .072 | 6.624 | 1 | .010 | 1.204 | 1.045 | 1.388 |
|  | [ATciclo=3] | .126 | .065 | 3.711 | 1 | .054 | 1.134 | .998 | 1.288 |
|  | [ATciclo=4] | .142 | .054 | 6.990 | 1 | .008 | 1.152 | 1.037 | 1.280 |
|  | [ATciclo=5] | 0^b^ | . | . | 0 | . | . | . | . |
|  | [ATdisf=1] | .159 | .077 | 4.293 | 1 | .038 | 1.172 | 1.009 | 1.361 |
|  | [ATdisf=2] | .183 | .073 | 6.204 | 1 | .013 | 1.200 | 1.040 | 1.386 |
|  | [ATdisf=3] | .196 | .064 | 9.478 | 1 | .002 | 1.216 | 1.074 | 1.377 |
|  | [ATdisf=4] | .125 | .050 | 6.257 | 1 | .012 | 1.133 | 1.027 | 1.250 |
|  | [ATdisf=5] | 0^b^ | . | . | 0 | . | . | . | . |
|  | [ATeut=1] | -.187 | .075 | 6.268 | 1 | .012 | .830 | .717 | .960 |
|  | [ATeut=2] | -.052 | .073 | .507 | 1 | .476 | .949 | .822 | 1.096 |
|  | [ATeut=3] | .024 | .075 | .106 | 1 | .744 | 1.025 | .885 | 1.186 |
|  | [ATeut=4] | .041 | .070 | .341 | 1 | .559 | 1.042 | .908 | 1.195 |
|  | [ATeut=5] | 0^b^ | . | . | 0 | . | . | . | . |
|  | [ATirrit=1] | -.004 | .076 | .003 | 1 | .954 | .996 | .857 | 1.156 |
|  | [ATirrit=2] | .103 | .064 | 2.588 | 1 | .108 | 1.109 | .978 | 1.257 |
|  | [ATirrit=3] | .118 | .054 | 4.806 | 1 | .028 | 1.125 | 1.013 | 1.251 |
|  | [ATirrit=4] | .143 | .049 | 8.497 | 1 | .004 | 1.153 | 1.048 | 1.269 |
|  | [ATirrit=5] | 0^b^ | . | . | 0 | . | . | . | . |
|  | [ATvolat=1] | .104 | .069 | 2.283 | 1 | .131 | 1.109 | .970 | 1.268 |
|  | [ATvolat=2] | .115 | .068 | 2.837 | 1 | .092 | 1.121 | .981 | 1.282 |
|  | [ATvolat=3] | .051 | .065 | .624 | 1 | .429 | 1.053 | .927 | 1.195 |
|  | [ATvolat=4] | .057 | .058 | .972 | 1 | .324 | 1.059 | .945 | 1.187 |
|  | [ATvolat=5] | 0^b^ | . | . | 0 | . | . | . | . |
|  | [ATdesin=1] | .047 | .065 | .510 | 1 | .475 | 1.048 | .922 | 1.191 |
|  | [ATdesin=2] | .022 | .065 | .114 | 1 | .736 | 1.022 | .900 | 1.160 |
|  | [ATdesin=3] | .017 | .060 | .079 | 1 | .779 | 1.017 | .904 | 1.145 |
|  | [ATdesin=4] | .027 | .054 | .256 | 1 | .613 | 1.028 | .925 | 1.142 |
|  | [ATdesin=5] | 0^b^ | . | . | 0 | . | . | . | . |
|  | [AThipertim=1] | .057 | .073 | .599 | 1 | .439 | 1.058 | .917 | 1.222 |
|  | [AThipertim=2] | .162 | .071 | 5.253 | 1 | .022 | 1.176 | 1.024 | 1.351 |
|  | [AThipertim=3] | .138 | .068 | 4.158 | 1 | .041 | 1.148 | 1.005 | 1.312 |
|  | [AThipertim=4] | .084 | .064 | 1.703 | 1 | .192 | 1.088 | .959 | 1.234 |
|  | [AThipertim=5] | 0^b^ | . | . | 0 | . | . | . | . |
|  | [ATCeuf=1] | .255 | .064 | 15.680 | 1 | .000 | 1.291 | 1.137 | 1.464 |
|  | [ATCeuf=2] | .242 | .063 | 14.609 | 1 | .000 | 1.274 | 1.125 | 1.442 |
|  | [ATCeuf=3] | .247 | .060 | 17.049 | 1 | .000 | 1.280 | 1.138 | 1.438 |
|  | [ATCeuf=4] | .078 | .055 | 1.969 | 1 | .161 | 1.081 | .970 | 1.204 |
|  | [ATCeuf=5] | 0^b^ | . | . | 0 | . | . | . | . |
|  | [sex=1] | -.176 | .039 | 20.824 | 1 | .000 | .839 | .778 | .904 |
|  | [sex=2] | 0^b^ | . | . | 0 | . | . | . | . |

ATdep: Affective temperament depressive; ATans: Affective temperament avoidant; ATapat: Affective temperament apathetic; ATobses: Affective temperament obsessive; ATciclo: Affective temperament cyclothymic; ATdisf: Affective temperament dysphoric; ATeut: Affective temperament euthymic; ATirrit: Affective temperament irritable; ATvolat: Affective temperament volatile; ATdesin: Affective temperament disinhibited; AThipertim: Affective temperament hyperthymic; ATCeuf: Affective temperament euphoric; Sex=1: Men; Sex=2: Women.

| **Table 3**  Laxatives Prediction | | | | | | | | | |
| --- | --- | --- | --- | --- | --- | --- | --- | --- | --- |
| Laxatives | | B | Standard error | Wald | gl | Sig. | Exp(B) | 95% Confidence interval Exp(B) | |
|  |  |  |  |  |  |  |  | Lower level | Upper level |
| Never | Interception | 1.636 | .137 | 142.820 | 1 | .000 |  |  |  |
|  | [ATdep=1] | .493 | .090 | 29.691 | 1 | .000 | 1.637 | 1.371 | 1.954 |
|  | [ATdep=2] | .405 | .083 | 23.930 | 1 | .000 | 1.499 | 1.274 | 1.762 |
|  | [ATdep=3] | .229 | .071 | 10.318 | 1 | .001 | 1.257 | 1.093 | 1.445 |
|  | [ATdep=4] | .247 | .064 | 15.033 | 1 | .000 | 1.280 | 1.130 | 1.450 |
|  | [ATdep=5] | 0^b^ | . | . | 0 | . | . | . | . |
|  | [ATans=1] | -.191 | .081 | 5.508 | 1 | .019 | .826 | .705 | .969 |
|  | [ATans=2] | .061 | .077 | .616 | 1 | .433 | 1.063 | .913 | 1.236 |
|  | [ATans=3] | -.105 | .068 | 2.388 | 1 | .122 | .901 | .789 | 1.028 |
|  | [ATans=4] | .019 | .062 | .097 | 1 | .755 | 1.020 | .902 | 1.153 |
|  | [ATans=5] | 0^b^ | . | . | 0 | . | . | . | . |
|  | [ATapat=1] | .061 | .083 | .537 | 1 | .464 | 1.063 | .903 | 1.252 |
|  | [ATapat=2] | -.014 | .081 | .030 | 1 | .863 | .986 | .842 | 1.155 |
|  | [ATapat=3] | -.001 | .077 | .000 | 1 | .993 | .999 | .860 | 1.162 |
|  | [ATapat=4] | .112 | .073 | 2.348 | 1 | .125 | 1.118 | .969 | 1.291 |
|  | [ATapat=5] | 0^b^ | . | . | 0 | . | . | . | . |
|  | [ATobses=1] | .336 | .078 | 18.348 | 1 | .000 | 1.399 | 1.200 | 1.632 |
|  | [ATobses=2] | .153 | .074 | 4.239 | 1 | .039 | 1.165 | 1.007 | 1.348 |
|  | [ATobses=3] | .249 | .071 | 12.364 | 1 | .000 | 1.283 | 1.116 | 1.474 |
|  | [ATobses=4] | .185 | .062 | 9.089 | 1 | .003 | 1.204 | 1.067 | 1.358 |
|  | [ATobses=5] | 0^b^ | . | . | 0 | . | . | . | . |
|  | [ATciclo=1] | .347 | .101 | 11.741 | 1 | .001 | 1.415 | 1.160 | 1.726 |
|  | [ATciclo=2] | .199 | .096 | 4.293 | 1 | .038 | 1.220 | 1.011 | 1.474 |
|  | [ATciclo=3] | .123 | .081 | 2.294 | 1 | .130 | 1.131 | .964 | 1.327 |
|  | [ATciclo=4] | .188 | .068 | 7.702 | 1 | .006 | 1.206 | 1.057 | 1.377 |
|  | [ATciclo=5] | 0^b^ | . | . | 0 | . | . | . | . |
|  | [ATdisf=1] | .357 | .103 | 11.908 | 1 | .001 | 1.429 | 1.167 | 1.750 |
|  | [ATdisf=2] | .457 | .102 | 19.933 | 1 | .000 | 1.579 | 1.292 | 1.930 |
|  | [ATdisf=3] | .195 | .079 | 6.120 | 1 | .013 | 1.216 | 1.041 | 1.419 |
|  | [ATdisf=4] | .196 | .062 | 9.871 | 1 | .002 | 1.216 | 1.076 | 1.374 |
|  | [ATdisf=5] | 0^b^ | . | . | 0 | . | . | . | . |
|  | [ATeut=1] | -.282 | .103 | 7.537 | 1 | .006 | .754 | .617 | .922 |
|  | [ATeut=2] | -.009 | .105 | .008 | 1 | .931 | .991 | .807 | 1.217 |
|  | [ATeut=3] | -.004 | .107 | .002 | 1 | .969 | .996 | .807 | 1.229 |
|  | [ATeut=4] | -.123 | .105 | 1.367 | 1 | .242 | .885 | .720 | 1.087 |
|  | [ATeut=5] | 0^b^ | . | . | 0 | . | . | . | . |
|  | [ATirrit=1] | -.040 | .091 | .189 | 1 | .663 | .961 | .804 | 1.149 |
|  | [ATirrit=2] | .156 | .084 | 3.415 | 1 | .065 | 1.169 | .991 | 1.379 |
|  | [ATirrit=3] | .079 | .067 | 1.370 | 1 | .242 | 1.082 | .948 | 1.235 |
|  | [ATirrit=4] | .018 | .060 | .091 | 1 | .763 | 1.018 | .905 | 1.145 |
|  | [ATirrit=5] | 0^b^ | . | . | 0 | . | . | . | . |
|  | [ATvolat=1] | .137 | .083 | 2.693 | 1 | .101 | 1.147 | .974 | 1.350 |
|  | [ATvolat=2] | .029 | .083 | .126 | 1 | .722 | 1.030 | .875 | 1.212 |
|  | [ATvolat=3] | .187 | .080 | 5.497 | 1 | .019 | 1.206 | 1.031 | 1.410 |
|  | [ATvolat=4] | .075 | .070 | 1.161 | 1 | .281 | 1.078 | .940 | 1.236 |
|  | [ATvolat=5] | 0^b^ | . | . | 0 | . | . | . | . |
|  | [ATdesin=1] | .036 | .079 | .210 | 1 | .647 | 1.037 | .888 | 1.210 |
|  | [ATdesin=2] | .228 | .083 | 7.635 | 1 | .006 | 1.256 | 1.069 | 1.477 |
|  | [ATdesin=3] | .064 | .074 | .764 | 1 | .382 | 1.067 | .923 | 1.232 |
|  | [ATdesin=4] | .141 | .067 | 4.429 | 1 | .035 | 1.151 | 1.010 | 1.312 |
|  | [ATdesin=5] | 0^b^ | . | . | 0 | . | . | . | . |
|  | [AThipertim=1] | -.289 | .100 | 8.414 | 1 | .004 | .749 | .616 | .910 |
|  | [AThipertim=2] | -.139 | .100 | 1.931 | 1 | .165 | .870 | .716 | 1.059 |
|  | [AThipertim=3] | -.057 | .098 | .340 | 1 | .560 | .944 | .778 | 1.145 |
|  | [AThipertim=4] | -.106 | .097 | 1.202 | 1 | .273 | .899 | .744 | 1.087 |
|  | [AThipertim=5] | 0^b^ | . | . | 0 | . | . | . | . |
|  | [ATCeuf=1] | .406 | .077 | 27.713 | 1 | .000 | 1.501 | 1.290 | 1.746 |
|  | [ATCeuf=2] | .486 | .082 | 35.284 | 1 | .000 | 1.625 | 1.385 | 1.908 |
|  | [ATCeuf=3] | .304 | .074 | 16.826 | 1 | .000 | 1.355 | 1.172 | 1.567 |
|  | [ATCeuf=4] | .126 | .069 | 3.297 | 1 | .069 | 1.134 | .990 | 1.299 |
|  | [ATCeuf=5] | 0^b^ | . | . | 0 | . | . | . | . |
|  | [sex=1] | 2.122 | .097 | 474.166 | 1 | .000 | 8.345 | 6.894 | 10.101 |
|  | [sex=2] | 0^b^ | . | . | 0 | . | . | . | . |
| Sometimes | Interception | .615 | .151 | 16.651 | 1 | .000 |  |  |  |
|  | [ATdep=1] | .309 | .100 | 9.555 | 1 | .002 | 1.362 | 1.120 | 1.656 |
|  | [ATdep=2] | .303 | .092 | 10.976 | 1 | .001 | 1.355 | 1.132 | 1.621 |
|  | [ATdep=3] | .259 | .079 | 10.702 | 1 | .001 | 1.296 | 1.110 | 1.514 |
|  | [ATdep=4] | .227 | .071 | 10.139 | 1 | .001 | 1.255 | 1.091 | 1.443 |
|  | [ATdep=5] | 0^b^ | . | . | 0 | . | . | . | . |
|  | [ATans=1] | -.154 | .090 | 2.935 | 1 | .087 | .857 | .719 | 1.022 |
|  | [ATans=2] | .007 | .085 | .006 | 1 | .936 | 1.007 | .852 | 1.189 |
|  | [ATans=3] | -.143 | .075 | 3.655 | 1 | .056 | .866 | .748 | 1.004 |
|  | [ATans=4] | -.027 | .069 | .147 | 1 | .701 | .974 | .851 | 1.115 |
|  | [ATans=5] | 0^b^ | . | . | 0 | . | . | . | . |
|  | [ATapat=1] | .138 | .093 | 2.204 | 1 | .138 | 1.148 | .957 | 1.376 |
|  | [ATapat=2] | .023 | .090 | .066 | 1 | .797 | 1.023 | .858 | 1.221 |
|  | [ATapat=3] | .055 | .086 | .410 | 1 | .522 | 1.057 | .893 | 1.250 |
|  | [ATapat=4] | .066 | .082 | .654 | 1 | .419 | 1.068 | .910 | 1.254 |
|  | [ATapat=5] | 0^b^ | . | . | 0 | . | . | . | . |
|  | [ATobses=1] | .289 | .086 | 11.161 | 1 | .001 | 1.335 | 1.127 | 1.582 |
|  | [ATobses=2] | .023 | .083 | .077 | 1 | .782 | 1.023 | .870 | 1.203 |
|  | [ATobses=3] | .169 | .078 | 4.677 | 1 | .031 | 1.184 | 1.016 | 1.380 |
|  | [ATobses=4] | .111 | .068 | 2.664 | 1 | .103 | 1.117 | .978 | 1.276 |
|  | [ATobses=5] | 0^b^ | . | . | 0 | . | . | . | . |
|  | [ATciclo=1] | .333 | .110 | 9.108 | 1 | .003 | 1.395 | 1.124 | 1.731 |
|  | [ATciclo=2] | .278 | .105 | 7.057 | 1 | .008 | 1.321 | 1.076 | 1.622 |
|  | [ATciclo=3] | .180 | .089 | 4.055 | 1 | .044 | 1.197 | 1.005 | 1.427 |
|  | [ATciclo=4] | .161 | .075 | 4.654 | 1 | .031 | 1.175 | 1.015 | 1.361 |
|  | [ATciclo=5] | 0^b^ | . | . | 0 | . | . | . | . |
|  | [ATdisf=1] | .054 | .113 | .229 | 1 | .633 | 1.056 | .846 | 1.317 |
|  | [ATdisf=2] | .222 | .111 | 3.983 | 1 | .046 | 1.248 | 1.004 | 1.552 |
|  | [ATdisf=3] | -.013 | .087 | .022 | 1 | .882 | .987 | .832 | 1.171 |
|  | [ATdisf=4] | .067 | .069 | .944 | 1 | .331 | 1.069 | .934 | 1.224 |
|  | [ATdisf=5] | 0^b^ | . | . | 0 | . | . | . | . |
|  | [ATeut=1] | -.278 | .112 | 6.156 | 1 | .013 | .757 | .608 | .943 |
|  | [ATeut=2] | -.103 | .114 | .824 | 1 | .364 | .902 | .722 | 1.127 |
|  | [ATeut=3] | -.078 | .116 | .451 | 1 | .502 | .925 | .737 | 1.161 |
|  | [ATeut=4] | -.145 | .113 | 1.641 | 1 | .200 | .865 | .693 | 1.080 |
|  | [ATeut=5] | 0^b^ | . | . | 0 | . | . | . | . |
|  | [ATirrit=1] | -.099 | .101 | .951 | 1 | .330 | .906 | .743 | 1.105 |
|  | [ATirrit=2] | .089 | .092 | .916 | 1 | .338 | 1.093 | .911 | 1.310 |
|  | [ATirrit=3] | .005 | .075 | .004 | 1 | .949 | 1.005 | .868 | 1.163 |
|  | [ATirrit=4] | .003 | .066 | .002 | 1 | .964 | 1.003 | .881 | 1.142 |
|  | [ATirrit=5] | 0^b^ | . | . | 0 | . | . | . | . |
|  | [ATvolat=1] | .017 | .093 | .035 | 1 | .852 | 1.017 | .848 | 1.220 |
|  | [ATvolat=2] | -.089 | .092 | .929 | 1 | .335 | .915 | .763 | 1.096 |
|  | [ATvolat=3] | .128 | .089 | 2.085 | 1 | .149 | 1.136 | .955 | 1.352 |
|  | [ATvolat=4] | .075 | .078 | .938 | 1 | .333 | 1.078 | .926 | 1.255 |
|  | [ATvolat=5] | 0^b^ | . | . | 0 | . | . | . | . |
|  | [ATdesin=1] | .114 | .087 | 1.713 | 1 | .191 | 1.121 | .945 | 1.331 |
|  | [ATdesin=2] | .219 | .091 | 5.735 | 1 | .017 | 1.244 | 1.040 | 1.488 |
|  | [ATdesin=3] | .092 | .082 | 1.263 | 1 | .261 | 1.097 | .934 | 1.288 |
|  | [ATdesin=4] | .167 | .074 | 5.044 | 1 | .025 | 1.181 | 1.021 | 1.366 |
|  | [ATdesin=5] | 0^b^ | . | . | 0 | . | . | . | . |
|  | [AThipertim=1] | -.236 | .109 | 4.661 | 1 | .031 | .790 | .637 | .978 |
|  | [AThipertim=2] | -.097 | .109 | .782 | 1 | .376 | .908 | .733 | 1.125 |
|  | [AThipertim=3] | -.012 | .107 | .012 | 1 | .913 | .988 | .801 | 1.219 |
|  | [AThipertim=4] | -.067 | .105 | .409 | 1 | .523 | .935 | .760 | 1.149 |
|  | [AThipertim=5] | 0^b^ | . | . | 0 | . | . | . | . |
|  | [ATCeuf=1] | .220 | .086 | 6.617 | 1 | .010 | 1.246 | 1.054 | 1.474 |
|  | [ATCeuf=2] | .261 | .090 | 8.404 | 1 | .004 | 1.299 | 1.088 | 1.550 |
|  | [ATCeuf=3] | .151 | .082 | 3.399 | 1 | .065 | 1.163 | .990 | 1.366 |
|  | [ATCeuf=4] | .023 | .077 | .090 | 1 | .764 | 1.023 | .880 | 1.190 |
|  | [ATCeuf=5] | 0^b^ | . | . | 0 | . | . | . | . |
|  | [sex=1] | .855 | .104 | 67.894 | 1 | .000 | 2.352 | 1.919 | 2.883 |
|  | [sex=2] | 0^b^ | . | . | 0 | . | . | . | . |

ATdep: Affective temperament depressive; ATans: Affective temperament avoidant; ATapat: Affective temperament apathetic; ATobses: Affective temperament obsessive; ATciclo: Affective temperament cyclothymic; ATdisf: Affective temperament dysphoric; ATeut: Affective temperament euthymic; ATirrit: Affective temperament irritable; ATvolat: Affective temperament volatile; ATdesin: Affective temperament disinhibited; AThipertim: Affective temperament hyperthymic; ATCeuf: Affective temperament euphoric; Sex=1: Men; Sex=2: Women.

| \| **Table 4**  Diuretics Prediction \| \| --- \| | | | | | | | | | |
| --- | --- | --- | --- | --- | --- | --- | --- | --- | --- | --- |
| Diuretics | | B | Standard error | Wald | gl | Sig. | Exp(B) | 95% Confidence interval Exp(B) | |
|  |  |  |  |  |  |  |  | Lower level | Upper level |
| Never | Interception | 1.721 | .148 | 134.592 | 1 | .000 |  |  |  |
|  | [ATdep=1] | .542 | .103 | 27.895 | 1 | .000 | 1.720 | 1.407 | 2.104 |
|  | [ATdep=2] | .339 | .093 | 13.334 | 1 | .000 | 1.403 | 1.170 | 1.683 |
|  | [ATdep=3] | .189 | .081 | 5.471 | 1 | .019 | 1.208 | 1.031 | 1.415 |
|  | [ATdep=4] | .207 | .073 | 7.976 | 1 | .005 | 1.230 | 1.065 | 1.419 |
|  | [ATdep=5] | 0^b^ | . | . | 0 | . | . | . | . |
|  | [ATans=1] | -.245 | .090 | 7.333 | 1 | .007 | .783 | .656 | .935 |
|  | [ATans=2] | -.001 | .086 | .000 | 1 | .992 | .999 | .843 | 1.184 |
|  | [ATans=3] | -.156 | .076 | 4.149 | 1 | .042 | .856 | .737 | .994 |
|  | [ATans=4] | .041 | .072 | .329 | 1 | .566 | 1.042 | .905 | 1.200 |
|  | [ATans=5] | 0^b^ | . | . | 0 | . | . | . | . |
|  | [ATapat=1] | -.031 | .094 | .105 | 1 | .745 | .970 | .806 | 1.167 |
|  | [ATapat=2] | -.079 | .092 | .749 | 1 | .387 | .924 | .772 | 1.106 |
|  | [ATapat=3] | -.025 | .089 | .079 | 1 | .778 | .975 | .820 | 1.160 |
|  | [ATapat=4] | .071 | .084 | .710 | 1 | .399 | 1.073 | .910 | 1.265 |
|  | [ATapat=5] | 0^b^ | . | . | 0 | . | . | . | . |
|  | [ATobses=1] | .280 | .088 | 10.143 | 1 | .001 | 1.324 | 1.114 | 1.573 |
|  | [ATobses=2] | .138 | .085 | 2.642 | 1 | .104 | 1.148 | .972 | 1.355 |
|  | [ATobses=3] | .286 | .082 | 12.166 | 1 | .000 | 1.331 | 1.133 | 1.562 |
|  | [ATobses=4] | .121 | .069 | 3.085 | 1 | .079 | 1.129 | .986 | 1.292 |
|  | [ATobses=5] | 0^b^ | . | . | 0 | . | . | . | . |
|  | [ATciclo=1] | .462 | .116 | 15.871 | 1 | .000 | 1.587 | 1.265 | 1.992 |
|  | [ATciclo=2] | .291 | .111 | 6.837 | 1 | .009 | 1.338 | 1.076 | 1.664 |
|  | [ATciclo=3] | .123 | .092 | 1.785 | 1 | .182 | 1.131 | .944 | 1.356 |
|  | [ATciclo=4] | .116 | .075 | 2.355 | 1 | .125 | 1.123 | .968 | 1.302 |
|  | [ATciclo=5] | 0^b^ | . | . | 0 | . | . | . | . |
|  | [ATdisf=1] | .319 | .116 | 7.604 | 1 | .006 | 1.376 | 1.097 | 1.727 |
|  | [ATdisf=2] | .393 | .114 | 11.907 | 1 | .001 | 1.481 | 1.185 | 1.851 |
|  | [ATdisf=3] | .328 | .093 | 12.342 | 1 | .000 | 1.389 | 1.156 | 1.668 |
|  | [ATdisf=4] | .147 | .070 | 4.428 | 1 | .035 | 1.159 | 1.010 | 1.329 |
|  | [ATdisf=5] | 0^b^ | . | . | 0 | . | . | . | . |
|  | [ATeut=1] | -.129 | .112 | 1.339 | 1 | .247 | .879 | .706 | 1.094 |
|  | [ATeut=2] | .162 | .114 | 1.994 | 1 | .158 | 1.175 | .939 | 1.471 |
|  | [ATeut=3] | .065 | .116 | .317 | 1 | .574 | 1.067 | .851 | 1.339 |
|  | [ATeut=4] | .048 | .115 | .174 | 1 | .677 | 1.049 | .837 | 1.315 |
|  | [ATeut=5] | 0^b^ | . | . | 0 | . | . | . | . |
|  | [ATirrit=1] | .035 | .107 | .107 | 1 | .744 | 1.035 | .840 | 1.276 |
|  | [ATirrit=2] | .103 | .095 | 1.172 | 1 | .279 | 1.108 | .920 | 1.335 |
|  | [ATirrit=3] | .054 | .076 | .505 | 1 | .477 | 1.056 | .909 | 1.226 |
|  | [ATirrit=4] | .027 | .068 | .159 | 1 | .690 | 1.027 | .900 | 1.173 |
|  | [ATirrit=5] | 0^b^ | . | . | 0 | . | . | . | . |
|  | [ATvolat=1] | .174 | .094 | 3.433 | 1 | .064 | 1.190 | .990 | 1.431 |
|  | [ATvolat=2] | .192 | .096 | 4.021 | 1 | .045 | 1.212 | 1.004 | 1.462 |
|  | [ATvolat=3] | .150 | .089 | 2.877 | 1 | .090 | 1.162 | .977 | 1.383 |
|  | [ATvolat=4] | .173 | .080 | 4.728 | 1 | .030 | 1.189 | 1.017 | 1.390 |
|  | [ATvolat=5] | 0^b^ | . | . | 0 | . | . | . | . |
|  | [ATdesin=1] | -.001 | .089 | .000 | 1 | .994 | .999 | .839 | 1.190 |
|  | [ATdesin=2] | .139 | .093 | 2.231 | 1 | .135 | 1.149 | .957 | 1.379 |
|  | [ATdesin=3] | .108 | .085 | 1.605 | 1 | .205 | 1.114 | .943 | 1.317 |
|  | [ATdesin=4] | .069 | .075 | .861 | 1 | .353 | 1.072 | .926 | 1.241 |
|  | [ATdesin=5] | 0^b^ | . | . | 0 | . | . | . | . |
|  | [AThipertim=1] | -.009 | .108 | .006 | 1 | .936 | .991 | .802 | 1.225 |
|  | [AThipertim=2] | .061 | .108 | .321 | 1 | .571 | 1.063 | .860 | 1.313 |
|  | [AThipertim=3] | .063 | .105 | .362 | 1 | .548 | 1.065 | .867 | 1.309 |
|  | [AThipertim=4] | .010 | .103 | .010 | 1 | .920 | 1.010 | .825 | 1.237 |
|  | [AThipertim=5] | 0^b^ | . | . | 0 | . | . | . | . |
|  | [ATCeuf=1] | .414 | .087 | 22.714 | 1 | .000 | 1.513 | 1.276 | 1.794 |
|  | [ATCeuf=2] | .500 | .092 | 29.560 | 1 | .000 | 1.649 | 1.377 | 1.976 |
|  | [ATCeuf=3] | .381 | .084 | 20.537 | 1 | .000 | 1.464 | 1.241 | 1.726 |
|  | [ATCeuf=4] | .160 | .077 | 4.295 | 1 | .038 | 1.173 | 1.009 | 1.364 |
|  | [ATCeuf=5] | 0^b^ | . | . | 0 | . | . | . | . |
|  | [sex=1] | 1.744 | .096 | 328.852 | 1 | .000 | 5.721 | 4.738 | 6.907 |
|  | [sex=2] | 0^b^ | . | . | 0 | . | . | . | . |
| Sometimes | Interception | .545 | .162 | 11.277 | 1 | .001 |  |  |  |
|  | [ATdep=1] | .407 | .112 | 13.328 | 1 | .000 | 1.503 | 1.208 | 1.870 |
|  | [ATdep=2] | .271 | .101 | 7.140 | 1 | .008 | 1.311 | 1.075 | 1.599 |
|  | [ATdep=3] | .140 | .089 | 2.486 | 1 | .115 | 1.151 | .967 | 1.370 |
|  | [ATdep=4] | .145 | .081 | 3.244 | 1 | .072 | 1.157 | .987 | 1.355 |
|  | [ATdep=5] | 0^b^ | . | . | 0 | . | . | . | . |
|  | [ATans=1] | -.238 | .099 | 5.754 | 1 | .016 | .788 | .649 | .957 |
|  | [ATans=2] | -.066 | .094 | .489 | 1 | .485 | .936 | .779 | 1.126 |
|  | [ATans=3] | -.153 | .084 | 3.354 | 1 | .067 | .858 | .728 | 1.011 |
|  | [ATans=4] | .037 | .078 | .225 | 1 | .636 | 1.038 | .890 | 1.210 |
|  | [ATans=5] | 0^b^ | . | . | 0 | . | . | . | . |
|  | [ATapat=1] | .097 | .104 | .873 | 1 | .350 | 1.102 | .899 | 1.350 |
|  | [ATapat=2] | .023 | .101 | .054 | 1 | .816 | 1.024 | .840 | 1.248 |
|  | [ATapat=3] | .085 | .098 | .758 | 1 | .384 | 1.089 | .899 | 1.318 |
|  | [ATapat=4] | .073 | .093 | .622 | 1 | .430 | 1.076 | .897 | 1.291 |
|  | [ATapat=5] | 0^b^ | . | . | 0 | . | . | . | . |
|  | [ATobses=1] | .185 | .097 | 3.679 | 1 | .055 | 1.203 | .996 | 1.454 |
|  | [ATobses=2] | .039 | .093 | .174 | 1 | .677 | 1.039 | .867 | 1.247 |
|  | [ATobses=3] | .247 | .089 | 7.748 | 1 | .005 | 1.280 | 1.076 | 1.523 |
|  | [ATobses=4] | .124 | .075 | 2.746 | 1 | .097 | 1.132 | .978 | 1.312 |
|  | [ATobses=5] | 0^b^ | . | . | 0 | . | . | . | . |
|  | [ATciclo=1] | .489 | .124 | 15.524 | 1 | .000 | 1.631 | 1.279 | 2.081 |
|  | [ATciclo=2] | .442 | .119 | 13.799 | 1 | .000 | 1.556 | 1.232 | 1.965 |
|  | [ATciclo=3] | .238 | .100 | 5.653 | 1 | .017 | 1.268 | 1.043 | 1.543 |
|  | [ATciclo=4] | .148 | .082 | 3.231 | 1 | .072 | 1.160 | .987 | 1.363 |
|  | [ATciclo=5] | 0^b^ | . | . | 0 | . | . | . | . |
|  | [ATdisf=1] | -.044 | .125 | .124 | 1 | .724 | .957 | .748 | 1.223 |
|  | [ATdisf=2] | .112 | .122 | .834 | 1 | .361 | 1.118 | .880 | 1.421 |
|  | [ATdisf=3] | .127 | .101 | 1.577 | 1 | .209 | 1.135 | .931 | 1.384 |
|  | [ATdisf=4] | .047 | .076 | .377 | 1 | .539 | 1.048 | .902 | 1.217 |
|  | [ATdisf=5] | 0^b^ | . | . | 0 | . | . | . | . |
|  | [ATeut=1] | -.210 | .121 | 3.036 | 1 | .081 | .810 | .640 | 1.027 |
|  | [ATeut=2] | .068 | .123 | .304 | 1 | .581 | 1.070 | .841 | 1.362 |
|  | [ATeut=3] | -.005 | .124 | .002 | 1 | .968 | .995 | .780 | 1.269 |
|  | [ATeut=4] | .052 | .123 | .179 | 1 | .672 | 1.053 | .828 | 1.339 |
|  | [ATeut=5] | 0^b^ | . | . | 0 | . | . | . | . |
|  | [ATirrit=1] | .041 | .116 | .123 | 1 | .726 | 1.042 | .830 | 1.307 |
|  | [ATirrit=2] | .078 | .103 | .577 | 1 | .448 | 1.081 | .884 | 1.322 |
|  | [ATirrit=3] | -.007 | .083 | .008 | 1 | .930 | .993 | .844 | 1.168 |
|  | [ATirrit=4] | -.011 | .074 | .023 | 1 | .879 | .989 | .855 | 1.143 |
|  | [ATirrit=5] | 0^b^ | . | . | 0 | . | . | . | . |
|  | [ATvolat=1] | .123 | .103 | 1.436 | 1 | .231 | 1.131 | .925 | 1.384 |
|  | [ATvolat=2] | .116 | .105 | 1.226 | 1 | .268 | 1.123 | .914 | 1.379 |
|  | [ATvolat=3] | .101 | .097 | 1.075 | 1 | .300 | 1.106 | .914 | 1.339 |
|  | [ATvolat=4] | .173 | .088 | 3.900 | 1 | .048 | 1.189 | 1.001 | 1.411 |
|  | [ATvolat=5] | 0^b^ | . | . | 0 | . | . | . | . |
|  | [ATdesin=1] | .031 | .098 | .103 | 1 | .748 | 1.032 | .852 | 1.250 |
|  | [ATdesin=2] | .142 | .101 | 1.948 | 1 | .163 | 1.152 | .944 | 1.405 |
|  | [ATdesin=3] | .163 | .093 | 3.078 | 1 | .079 | 1.177 | .981 | 1.413 |
|  | [ATdesin=4] | .116 | .082 | 1.992 | 1 | .158 | 1.123 | .956 | 1.319 |
|  | [ATdesin=5] | 0^b^ | . | . | 0 | . | . | . | . |
|  | [AThipertim=1] | .024 | .117 | .043 | 1 | .836 | 1.025 | .814 | 1.290 |
|  | [AThipertim=2] | .105 | .117 | .816 | 1 | .366 | 1.111 | .884 | 1.397 |
|  | [AThipertim=3] | .045 | .113 | .157 | 1 | .692 | 1.046 | .837 | 1.306 |
|  | [AThipertim=4] | -.002 | .111 | .000 | 1 | .982 | .998 | .802 | 1.240 |
|  | [AThipertim=5] | 0^b^ | . | . | 0 | . | . | . | . |
|  | [ATCeuf=1] | .125 | .096 | 1.707 | 1 | .191 | 1.133 | .939 | 1.366 |
|  | [ATCeuf=2] | .246 | .100 | 6.015 | 1 | .014 | 1.279 | 1.051 | 1.556 |
|  | [ATCeuf=3] | .254 | .092 | 7.691 | 1 | .006 | 1.289 | 1.077 | 1.543 |
|  | [ATCeuf=4] | .113 | .084 | 1.782 | 1 | .182 | 1.119 | .949 | 1.321 |
|  | [ATCeuf=5] | 0^b^ | . | . | 0 | . | . | . | . |
|  | [sex=1] | .627 | .102 | 37.577 | 1 | .000 | 1.871 | 1.531 | 2.286 |
|  | [sex=2] | 0^b^ | . | . | 0 | . | . | . | . |

ATdep: Affective temperament depressive; ATans: Affective temperament avoidant; ATapat: Affective temperament apathetic; ATobses: Affective temperament obsessive; ATciclo: Affective temperament cyclothymic; ATdisf: Affective temperament dysphoric; ATeut: Affective temperament euthymic; ATirrit: Affective temperament irritable; ATvolat: Affective temperament volatile; ATdesin: Affective temperament disinhibited; AThipertim: Affective temperament hyperthymic; ATCeuf: Affective temperament euphoric; Sex=1: Men; Sex=2: Women.

| **Table 5**  Medication Prediction | | | | | | | | | |
| --- | --- | --- | --- | --- | --- | --- | --- | --- | --- |
| Medication | | B | Standard error | Wald | gl | Sig. | Exp(B) | 95% Confidence interval Exp(B) | |
|  |  |  |  |  |  |  |  | Lower level | Upper level |
| Never | Interception | 1.449 | .121 | 142.458 | 1 | .000 |  |  |  |
|  | [ATdep=1] | .411 | .080 | 26.233 | 1 | .000 | 1.508 | 1.289 | 1.765 |
|  | [ATdep=2] | .350 | .075 | 21.505 | 1 | .000 | 1.419 | 1.224 | 1.645 |
|  | [ATdep=3] | .283 | .068 | 17.513 | 1 | .000 | 1.327 | 1.162 | 1.514 |
|  | [ATdep=4] | .188 | .060 | 9.740 | 1 | .002 | 1.206 | 1.072 | 1.357 |
|  | [ATdep=5] | 0^b^ | . | . | 0 | . | . | . | . |
|  | [ATans=1] | -.140 | .074 | 3.529 | 1 | .060 | .870 | .752 | 1.006 |
|  | [ATans=2] | -.009 | .069 | .019 | 1 | .891 | .991 | .866 | 1.133 |
|  | [ATans=3] | -.056 | .063 | .792 | 1 | .373 | .946 | .837 | 1.069 |
|  | [ATans=4] | .044 | .058 | .572 | 1 | .450 | 1.045 | .933 | 1.170 |
|  | [ATans=5] | 0^b^ | . | . | 0 | . | . | . | . |
|  | [ATapat=1] | -.187 | .077 | 5.853 | 1 | .016 | .830 | .713 | .965 |
|  | [ATapat=2] | -.146 | .076 | 3.684 | 1 | .055 | .864 | .744 | 1.003 |
|  | [ATapat=3] | -.101 | .074 | 1.872 | 1 | .171 | .904 | .782 | 1.045 |
|  | [ATapat=4] | .014 | .070 | .041 | 1 | .840 | 1.014 | .883 | 1.165 |
|  | [ATapat=5] | 0^b^ | . | . | 0 | . | . | . | . |
|  | [ATobses=1] | .121 | .071 | 2.931 | 1 | .087 | 1.129 | .983 | 1.296 |
|  | [ATobses=2] | .179 | .070 | 6.526 | 1 | .011 | 1.197 | 1.043 | 1.373 |
|  | [ATobses=3] | .205 | .065 | 9.839 | 1 | .002 | 1.227 | 1.080 | 1.395 |
|  | [ATobses=4] | .108 | .056 | 3.747 | 1 | .053 | 1.114 | .999 | 1.242 |
|  | [ATobses=5] | 0^b^ | . | . | 0 | . | . | . | . |
|  | [ATciclo=1] | -.010 | .087 | .013 | 1 | .911 | .990 | .836 | 1.174 |
|  | [ATciclo=2] | .130 | .089 | 2.169 | 1 | .141 | 1.139 | .958 | 1.355 |
|  | [ATciclo=3] | -.115 | .072 | 2.527 | 1 | .112 | .891 | .774 | 1.027 |
|  | [ATciclo=4] | .036 | .062 | .341 | 1 | .559 | 1.037 | .919 | 1.170 |
|  | [ATciclo=5] | 0^b^ | . | . | 0 | . | . | . | . |
|  | [ATdisf=1] | .410 | .093 | 19.443 | 1 | .000 | 1.507 | 1.256 | 1.809 |
|  | [ATdisf=2] | .390 | .089 | 19.092 | 1 | .000 | 1.477 | 1.240 | 1.760 |
|  | [ATdisf=3] | .231 | .072 | 10.316 | 1 | .001 | 1.260 | 1.094 | 1.451 |
|  | [ATdisf=4] | .239 | .058 | 17.292 | 1 | .000 | 1.270 | 1.135 | 1.422 |
|  | [ATdisf=5] | 0^b^ | . | . | 0 | . | . | . | . |
|  | [ATeut=1] | -.450 | .091 | 24.328 | 1 | .000 | .638 | .533 | .762 |
|  | [ATeut=2] | -.237 | .092 | 6.667 | 1 | .010 | .789 | .659 | .944 |
|  | [ATeut=3] | -.256 | .092 | 7.698 | 1 | .006 | .774 | .646 | .927 |
|  | [ATeut=4] | -.187 | .091 | 4.213 | 1 | .040 | .829 | .694 | .992 |
|  | [ATeut=5] | 0^b^ | . | . | 0 | . | . | . | . |
|  | [ATirrit=1] | .252 | .088 | 8.110 | 1 | .004 | 1.286 | 1.082 | 1.530 |
|  | [ATirrit=2] | .265 | .076 | 12.141 | 1 | .000 | 1.304 | 1.123 | 1.514 |
|  | [ATirrit=3] | .256 | .062 | 16.811 | 1 | .000 | 1.291 | 1.143 | 1.459 |
|  | [ATirrit=4] | .168 | .055 | 9.486 | 1 | .002 | 1.183 | 1.063 | 1.317 |
|  | [ATirrit=5] | 0^b^ | . | . | 0 | . | . | . | . |
|  | [ATvolat=1] | .039 | .076 | .266 | 1 | .606 | 1.040 | .897 | 1.206 |
|  | [ATvolat=2] | .085 | .077 | 1.218 | 1 | .270 | 1.089 | .936 | 1.266 |
|  | [ATvolat=3] | .149 | .073 | 4.119 | 1 | .042 | 1.160 | 1.005 | 1.340 |
|  | [ATvolat=4] | .212 | .066 | 10.204 | 1 | .001 | 1.237 | 1.086 | 1.409 |
|  | [ATvolat=5] | 0^b^ | . | . | 0 | . | . | . | . |
|  | [ATdesin=1] | .125 | .073 | 2.919 | 1 | .088 | 1.133 | .982 | 1.308 |
|  | [ATdesin=2] | .101 | .074 | 1.842 | 1 | .175 | 1.106 | .956 | 1.279 |
|  | [ATdesin=3] | .069 | .068 | 1.035 | 1 | .309 | 1.072 | .938 | 1.225 |
|  | [ATdesin=4] | .074 | .061 | 1.486 | 1 | .223 | 1.077 | .956 | 1.214 |
|  | [ATdesin=5] | 0^b^ | . | . | 0 | . | . | . | . |
|  | [AThipertim=1] | .070 | .085 | .671 | 1 | .413 | 1.072 | .907 | 1.267 |
|  | [AThipertim=2] | .113 | .084 | 1.789 | 1 | .181 | 1.119 | .949 | 1.321 |
|  | [AThipertim=3] | .206 | .083 | 6.236 | 1 | .013 | 1.229 | 1.045 | 1.446 |
|  | [AThipertim=4] | .012 | .079 | .022 | 1 | .882 | 1.012 | .867 | 1.181 |
|  | [AThipertim=5] | 0^b^ | . | . | 0 | . | . | . | . |
|  | [ATCeuf=1] | .512 | .071 | 51.396 | 1 | .000 | 1.669 | 1.451 | 1.920 |
|  | [ATCeuf=2] | .561 | .075 | 56.521 | 1 | .000 | 1.752 | 1.514 | 2.028 |
|  | [ATCeuf=3] | .393 | .067 | 33.912 | 1 | .000 | 1.481 | 1.298 | 1.690 |
|  | [ATCeuf=4] | .133 | .062 | 4.612 | 1 | .032 | 1.142 | 1.012 | 1.289 |
|  | [ATCeuf=5] | 0^b^ | . | . | 0 | . | . | . | . |
|  | [sex=1] | 1.528 | .068 | 500.652 | 1 | .000 | 4.608 | 4.031 | 5.268 |
|  | [sex=2] | 0^b^ | . | . | 0 | . | . | . | . |
| Sometimes | Interception | .583 | .132 | 19.541 | 1 | .000 |  |  |  |
|  | [ATdep=1] | .256 | .087 | 8.632 | 1 | .003 | 1.292 | 1.089 | 1.533 |
|  | [ATdep=2] | .300 | .082 | 13.368 | 1 | .000 | 1.349 | 1.149 | 1.584 |
|  | [ATdep=3] | .249 | .074 | 11.386 | 1 | .001 | 1.282 | 1.110 | 1.482 |
|  | [ATdep=4] | .086 | .066 | 1.677 | 1 | .195 | 1.090 | .957 | 1.240 |
|  | [ATdep=5] | 0^b^ | . | . | 0 | . | . | . | . |
|  | [ATans=1] | -.063 | .081 | .604 | 1 | .437 | .939 | .802 | 1.100 |
|  | [ATans=2] | -.081 | .075 | 1.193 | 1 | .275 | .922 | .796 | 1.067 |
|  | [ATans=3] | -.103 | .068 | 2.273 | 1 | .132 | .902 | .789 | 1.031 |
|  | [ATans=4] | -.020 | .063 | .103 | 1 | .748 | .980 | .866 | 1.109 |
|  | [ATans=5] | 0^b^ | . | . | 0 | . | . | . | . |
|  | [ATapat=1] | .032 | .084 | .142 | 1 | .706 | 1.032 | .875 | 1.218 |
|  | [ATapat=2] | .012 | .083 | .022 | 1 | .882 | 1.012 | .860 | 1.192 |
|  | [ATapat=3] | .038 | .081 | .216 | 1 | .642 | 1.038 | .886 | 1.217 |
|  | [ATapat=4] | .055 | .077 | .511 | 1 | .475 | 1.057 | .908 | 1.230 |
|  | [ATapat=5] | 0^b^ | . | . | 0 | . | . | . | . |
|  | [ATobses=1] | .119 | .077 | 2.394 | 1 | .122 | 1.126 | .969 | 1.310 |
|  | [ATobses=2] | .092 | .076 | 1.452 | 1 | .228 | 1.096 | .944 | 1.274 |
|  | [ATobses=3] | .138 | .071 | 3.812 | 1 | .051 | 1.148 | .999 | 1.320 |
|  | [ATobses=4] | .107 | .060 | 3.140 | 1 | .076 | 1.113 | .989 | 1.253 |
|  | [ATobses=5] | 0^b^ | . | . | 0 | . | . | . | . |
|  | [ATciclo=1] | .187 | .093 | 4.060 | 1 | .044 | 1.206 | 1.005 | 1.447 |
|  | [ATciclo=2] | .346 | .095 | 13.374 | 1 | .000 | 1.413 | 1.174 | 1.701 |
|  | [ATciclo=3] | .090 | .078 | 1.315 | 1 | .251 | 1.094 | .938 | 1.275 |
|  | [ATciclo=4] | .127 | .067 | 3.569 | 1 | .059 | 1.135 | .995 | 1.295 |
|  | [ATciclo=5] | 0^b^ | . | . | 0 | . | . | . | . |
|  | [ATdisf=1] | .184 | .100 | 3.412 | 1 | .065 | 1.203 | .989 | 1.463 |
|  | [ATdisf=2] | .228 | .096 | 5.664 | 1 | .017 | 1.256 | 1.041 | 1.516 |
|  | [ATdisf=3] | .082 | .078 | 1.105 | 1 | .293 | 1.086 | .931 | 1.266 |
|  | [ATdisf=4] | .179 | .063 | 8.245 | 1 | .004 | 1.197 | 1.059 | 1.353 |
|  | [ATdisf=5] | 0^b^ | . | . | 0 | . | . | . | . |
|  | [ATeut=1] | -.429 | .098 | 19.202 | 1 | .000 | .651 | .537 | .789 |
|  | [ATeut=2] | -.322 | .098 | 10.718 | 1 | .001 | .725 | .598 | .879 |
|  | [ATeut=3] | -.303 | .099 | 9.400 | 1 | .002 | .739 | .609 | .897 |
|  | [ATeut=4] | -.163 | .097 | 2.839 | 1 | .092 | .850 | .703 | 1.027 |
|  | [ATeut=5] | 0^b^ | . | . | 0 | . | . | . | . |
|  | [ATirrit=1] | .221 | .095 | 5.369 | 1 | .021 | 1.247 | 1.035 | 1.504 |
|  | [ATirrit=2] | .181 | .082 | 4.845 | 1 | .028 | 1.199 | 1.020 | 1.409 |
|  | [ATirrit=3] | .174 | .068 | 6.610 | 1 | .010 | 1.190 | 1.042 | 1.359 |
|  | [ATirrit=4] | .146 | .059 | 5.985 | 1 | .014 | 1.157 | 1.029 | 1.300 |
|  | [ATirrit=5] | 0^b^ | . | . | 0 | . | . | . | . |
|  | [ATvolat=1] | -.079 | .083 | .913 | 1 | .339 | .924 | .786 | 1.087 |
|  | [ATvolat=2] | .002 | .084 | .001 | 1 | .980 | 1.002 | .850 | 1.182 |
|  | [ATvolat=3] | .110 | .080 | 1.905 | 1 | .168 | 1.117 | .955 | 1.306 |
|  | [ATvolat=4] | .134 | .073 | 3.411 | 1 | .065 | 1.144 | .992 | 1.319 |
|  | [ATvolat=5] | 0^b^ | . | . | 0 | . | . | . | . |
|  | [ATdesin=1] | .145 | .080 | 3.323 | 1 | .068 | 1.156 | .989 | 1.351 |
|  | [ATdesin=2] | .117 | .081 | 2.114 | 1 | .146 | 1.124 | .960 | 1.317 |
|  | [ATdesin=3] | .039 | .075 | .271 | 1 | .602 | 1.040 | .898 | 1.203 |
|  | [ATdesin=4] | .092 | .067 | 1.921 | 1 | .166 | 1.097 | .962 | 1.250 |
|  | [ATdesin=5] | 0^b^ | . | . | 0 | . | . | . | . |
|  | [AThipertim=1] | .081 | .093 | .756 | 1 | .385 | 1.084 | .904 | 1.300 |
|  | [AThipertim=2] | .137 | .091 | 2.247 | 1 | .134 | 1.147 | .959 | 1.372 |
|  | [AThipertim=3] | .218 | .089 | 5.987 | 1 | .014 | 1.244 | 1.044 | 1.481 |
|  | [AThipertim=4] | .051 | .085 | .357 | 1 | .550 | 1.052 | .890 | 1.243 |
|  | [AThipertim=5] | 0^b^ | . | . | 0 | . | . | . | . |
|  | [ATCeuf=1] | .260 | .078 | 11.164 | 1 | .001 | 1.297 | 1.114 | 1.511 |
|  | [ATCeuf=2] | .322 | .081 | 15.867 | 1 | .000 | 1.380 | 1.178 | 1.617 |
|  | [ATCeuf=3] | .187 | .074 | 6.462 | 1 | .011 | 1.206 | 1.044 | 1.393 |
|  | [ATCeuf=4] | .042 | .068 | .392 | 1 | .531 | 1.043 | .914 | 1.192 |
|  | [ATCeuf=5] | 0^b^ | . | . | 0 | . | . | . | . |
|  | [sex=1] | .530 | .073 | 52.248 | 1 | .000 | 1.698 | 1.471 | 1.961 |
|  | [sex=2] | 0^b^ | . | . | 0 | . | . | . | . |

ATdep: Affective temperament depressive; ATans: Affective temperament avoidant; ATapat: Affective temperament apathetic; ATobses: Affective temperament obsessive; ATciclo: Affective temperament cyclothymic; ATdisf: Affective temperament dysphoric; ATeut: Affective temperament euthymic; ATirrit: Affective temperament irritable; ATvolat: Affective temperament volatile; ATdesin: Affective temperament disinhibited; AThipertim: Affective temperament hyperthymic; ATCeuf: Affective temperament euphoric; Sex=1: Men; Sex=2: Women.

| **Table 6**  Vomiting Prediction | | | | | | | | | |
| --- | --- | --- | --- | --- | --- | --- | --- | --- | --- |
| Vomiting | | B | Standard error | Wald | gl | Sig. | Exp(B) | 95% Confidence interval Exp(B) | |
|  |  |  |  |  |  |  |  | Lower level | Upper level |
| Never | Interception | 2.528 | .185 | 187.032 | 1 | .000 |  |  |  |
|  | [ATdep=1] | .872 | .114 | 58.278 | 1 | .000 | 2.391 | 1.911 | 2.990 |
|  | [ATdep=2] | .678 | .098 | 48.088 | 1 | .000 | 1.970 | 1.626 | 2.386 |
|  | [ATdep=3] | .515 | .082 | 39.257 | 1 | .000 | 1.674 | 1.425 | 1.966 |
|  | [ATdep=4] | .419 | .071 | 35.046 | 1 | .000 | 1.520 | 1.323 | 1.747 |
|  | [ATdep=5] | 0^b^ | . | . | 0 | . | . | . | . |
|  | [ATans=1] | -.455 | .095 | 22.897 | 1 | .000 | .634 | .526 | .764 |
|  | [ATans=2] | -.372 | .087 | 18.243 | 1 | .000 | .689 | .581 | .818 |
|  | [ATans=3] | -.274 | .081 | 11.547 | 1 | .001 | .760 | .649 | .891 |
|  | [ATans=4] | -.096 | .075 | 1.630 | 1 | .202 | .909 | .784 | 1.053 |
|  | [ATans=5] | 0^b^ | . | . | 0 | . | . | . | . |
|  | [ATapat=1] | .095 | .098 | .941 | 1 | .332 | 1.100 | .908 | 1.332 |
|  | [ATapat=2] | .041 | .092 | .195 | 1 | .659 | 1.041 | .870 | 1.247 |
|  | [ATapat=3] | .057 | .086 | .432 | 1 | .511 | 1.058 | .893 | 1.254 |
|  | [ATapat=4] | .102 | .080 | 1.635 | 1 | .201 | 1.107 | .947 | 1.294 |
|  | [ATapat=5] | 0^b^ | . | . | 0 | . | . | . | . |
|  | [ATobses=1] | -.037 | .087 | .176 | 1 | .674 | .964 | .813 | 1.143 |
|  | [ATobses=2] | .002 | .088 | .001 | 1 | .979 | 1.002 | .844 | 1.190 |
|  | [ATobses=3] | .005 | .082 | .003 | 1 | .955 | 1.005 | .855 | 1.181 |
|  | [ATobses=4] | -.005 | .074 | .004 | 1 | .948 | .995 | .860 | 1.152 |
|  | [ATobses=5] | 0^b^ | . | . | 0 | . | . | . | . |
|  | [ATciclo=1] | .638 | .142 | 20.242 | 1 | .000 | 1.893 | 1.434 | 2.500 |
|  | [ATciclo=2] | .202 | .117 | 2.968 | 1 | .085 | 1.223 | .973 | 1.539 |
|  | [ATciclo=3] | .251 | .101 | 6.186 | 1 | .013 | 1.286 | 1.055 | 1.568 |
|  | [ATciclo=4] | .170 | .077 | 4.933 | 1 | .026 | 1.186 | 1.020 | 1.378 |
|  | [ATciclo=5] | 0^b^ | . | . | 0 | . | . | . | . |
|  | [ATdisf=1] | .310 | .129 | 5.740 | 1 | .017 | 1.364 | 1.058 | 1.757 |
|  | [ATdisf=2] | .397 | .122 | 10.611 | 1 | .001 | 1.487 | 1.171 | 1.887 |
|  | [ATdisf=3] | .310 | .097 | 10.265 | 1 | .001 | 1.363 | 1.128 | 1.647 |
|  | [ATdisf=4] | .265 | .072 | 13.435 | 1 | .000 | 1.303 | 1.131 | 1.502 |
|  | [ATdisf=5] | 0^b^ | . | . | 0 | . | . | . | . |
|  | [ATeut=1] | -.994 | .151 | 43.218 | 1 | .000 | .370 | .275 | .498 |
|  | [ATeut=2] | -.637 | .153 | 17.341 | 1 | .000 | .529 | .392 | .714 |
|  | [ATeut=3] | -.480 | .158 | 9.212 | 1 | .002 | .619 | .454 | .844 |
|  | [ATeut=4] | -.312 | .163 | 3.663 | 1 | .056 | .732 | .532 | 1.008 |
|  | [ATeut=5] | 0^b^ | . | . | 0 | . | . | . | . |
|  | [ATirrit=1] | -.122 | .107 | 1.285 | 1 | .257 | .885 | .718 | 1.093 |
|  | [ATirrit=2] | -.132 | .094 | 1.996 | 1 | .158 | .876 | .730 | 1.052 |
|  | [ATirrit=3] | -.035 | .079 | .199 | 1 | .656 | .965 | .827 | 1.127 |
|  | [ATirrit=4] | -.009 | .071 | .017 | 1 | .897 | .991 | .862 | 1.138 |
|  | [ATirrit=5] | 0^b^ | . | . | 0 | . | . | . | . |
|  | [ATvolat=1] | .476 | .102 | 21.733 | 1 | .000 | 1.610 | 1.318 | 1.967 |
|  | [ATvolat=2] | .230 | .098 | 5.482 | 1 | .019 | 1.259 | 1.038 | 1.527 |
|  | [ATvolat=3] | .197 | .089 | 4.838 | 1 | .028 | 1.217 | 1.022 | 1.450 |
|  | [ATvolat=4] | .013 | .075 | .030 | 1 | .862 | 1.013 | .874 | 1.174 |
|  | [ATvolat=5] | 0^b^ | . | . | 0 | . | . | . | . |
|  | [ATdesin=1] | .116 | .096 | 1.469 | 1 | .226 | 1.123 | .931 | 1.355 |
|  | [ATdesin=2] | .052 | .093 | .317 | 1 | .573 | 1.054 | .879 | 1.263 |
|  | [ATdesin=3] | .129 | .086 | 2.264 | 1 | .132 | 1.138 | .962 | 1.347 |
|  | [ATdesin=4] | .123 | .076 | 2.652 | 1 | .103 | 1.131 | .975 | 1.311 |
|  | [ATdesin=5] | 0^b^ | . | . | 0 | . | . | . | . |
|  | [AThipertim=1] | -.055 | .122 | .204 | 1 | .651 | .946 | .745 | 1.202 |
|  | [AThipertim=2] | -.022 | .122 | .032 | 1 | .858 | .978 | .770 | 1.243 |
|  | [AThipertim=3] | .083 | .123 | .454 | 1 | .501 | 1.086 | .854 | 1.382 |
|  | [AThipertim=4] | .142 | .126 | 1.267 | 1 | .260 | 1.153 | .900 | 1.477 |
|  | [AThipertim=5] | 0^b^ | . | . | 0 | . | . | . | . |
|  | [ATCeuf=1] | .483 | .091 | 28.355 | 1 | .000 | 1.621 | 1.357 | 1.936 |
|  | [ATCeuf=2] | .335 | .092 | 13.222 | 1 | .000 | 1.398 | 1.167 | 1.675 |
|  | [ATCeuf=3] | .326 | .087 | 14.146 | 1 | .000 | 1.386 | 1.169 | 1.642 |
|  | [ATCeuf=4] | .149 | .080 | 3.432 | 1 | .064 | 1.160 | .991 | 1.358 |
|  | [ATCeuf=5] | 0^b^ | . | . | 0 | . | . | . | . |
|  | [sex=1] | 1.676 | .100 | 283.115 | 1 | .000 | 5.342 | 4.395 | 6.494 |
|  | [sex=2] | 0^b^ | . | . | 0 | . | . | . | . |
| Sometimes | Interception | 1.050 | .205 | 26.332 | 1 | .000 |  |  |  |
|  | [ATdep=1] | .330 | .128 | 6.656 | 1 | .010 | 1.391 | 1.083 | 1.787 |
|  | [ATdep=2] | .279 | .110 | 6.449 | 1 | .011 | 1.322 | 1.066 | 1.641 |
|  | [ATdep=3] | .252 | .093 | 7.367 | 1 | .007 | 1.287 | 1.073 | 1.544 |
|  | [ATdep=4] | .223 | .080 | 7.747 | 1 | .005 | 1.250 | 1.068 | 1.463 |
|  | [ATdep=5] | 0^b^ | . | . | 0 | . | . | . | . |
|  | [ATans=1] | -.300 | .108 | 7.753 | 1 | .005 | .741 | .600 | .915 |
|  | [ATans=2] | -.218 | .098 | 4.972 | 1 | .026 | .804 | .664 | .974 |
|  | [ATans=3] | -.136 | .090 | 2.255 | 1 | .133 | .873 | .731 | 1.042 |
|  | [ATans=4] | -.151 | .085 | 3.171 | 1 | .075 | .860 | .729 | 1.015 |
|  | [ATans=5] | 0^b^ | . | . | 0 | . | . | . | . |
|  | [ATapat=1] | .022 | .111 | .038 | 1 | .845 | 1.022 | .822 | 1.271 |
|  | [ATapat=2] | .069 | .104 | .436 | 1 | .509 | 1.071 | .873 | 1.314 |
|  | [ATapat=3] | .158 | .098 | 2.599 | 1 | .107 | 1.171 | .967 | 1.418 |
|  | [ATapat=4] | .147 | .090 | 2.646 | 1 | .104 | 1.158 | .970 | 1.382 |
|  | [ATapat=5] | 0^b^ | . | . | 0 | . | . | . | . |
|  | [ATobses=1] | .112 | .098 | 1.309 | 1 | .253 | 1.119 | .923 | 1.356 |
|  | [ATobses=2] | .069 | .099 | .482 | 1 | .488 | 1.071 | .882 | 1.300 |
|  | [ATobses=3] | .008 | .093 | .008 | 1 | .929 | 1.008 | .840 | 1.211 |
|  | [ATobses=4] | .118 | .084 | 1.973 | 1 | .160 | 1.125 | .955 | 1.325 |
|  | [ATobses=5] | 0^b^ | . | . | 0 | . | . | . | . |
|  | [ATciclo=1] | .286 | .156 | 3.351 | 1 | .067 | 1.332 | .980 | 1.809 |
|  | [ATciclo=2] | .002 | .132 | .000 | 1 | .990 | 1.002 | .774 | 1.296 |
|  | [ATciclo=3] | .189 | .113 | 2.816 | 1 | .093 | 1.208 | .969 | 1.506 |
|  | [ATciclo=4] | .039 | .087 | .201 | 1 | .654 | 1.040 | .877 | 1.232 |
|  | [ATciclo=5] | 0^b^ | . | . | 0 | . | . | . | . |
|  | [ATdisf=1] | .022 | .145 | .022 | 1 | .881 | 1.022 | .769 | 1.359 |
|  | [ATdisf=2] | .209 | .135 | 2.377 | 1 | .123 | 1.232 | .945 | 1.606 |
|  | [ATdisf=3] | .156 | .108 | 2.076 | 1 | .150 | 1.169 | .945 | 1.445 |
|  | [ATdisf=4] | .108 | .081 | 1.757 | 1 | .185 | 1.114 | .950 | 1.307 |
|  | [ATdisf=5] | 0^b^ | . | . | 0 | . | . | . | . |
|  | [ATeut=1] | -.582 | .165 | 12.417 | 1 | .000 | .559 | .404 | .772 |
|  | [ATeut=2] | -.527 | .167 | 9.893 | 1 | .002 | .591 | .425 | .820 |
|  | [ATeut=3] | -.401 | .173 | 5.403 | 1 | .020 | .670 | .477 | .939 |
|  | [ATeut=4] | -.272 | .177 | 2.359 | 1 | .125 | .762 | .538 | 1.078 |
|  | [ATeut=5] | 0^b^ | . | . | 0 | . | . | . | . |
|  | [ATirrit=1] | -.118 | .122 | .930 | 1 | .335 | .889 | .700 | 1.129 |
|  | [ATirrit=2] | -.098 | .106 | .863 | 1 | .353 | .906 | .737 | 1.115 |
|  | [ATirrit=3] | .038 | .088 | .186 | 1 | .667 | 1.039 | .874 | 1.235 |
|  | [ATirrit=4] | .037 | .080 | .214 | 1 | .644 | 1.038 | .888 | 1.213 |
|  | [ATirrit=5] | 0^b^ | . | . | 0 | . | . | . | . |
|  | [ATvolat=1] | .030 | .115 | .066 | 1 | .797 | 1.030 | .822 | 1.292 |
|  | [ATvolat=2] | .045 | .111 | .165 | 1 | .684 | 1.046 | .842 | 1.299 |
|  | [ATvolat=3] | .002 | .101 | .000 | 1 | .983 | 1.002 | .823 | 1.221 |
|  | [ATvolat=4] | -.081 | .085 | .903 | 1 | .342 | .922 | .780 | 1.090 |
|  | [ATvolat=5] | 0^b^ | . | . | 0 | . | . | . | . |
|  | [ATdesin=1] | .049 | .108 | .205 | 1 | .650 | 1.050 | .849 | 1.299 |
|  | [ATdesin=2] | -.023 | .105 | .046 | 1 | .829 | .978 | .796 | 1.201 |
|  | [ATdesin=3] | .125 | .097 | 1.658 | 1 | .198 | 1.133 | .937 | 1.370 |
|  | [ATdesin=4] | .152 | .085 | 3.209 | 1 | .073 | 1.164 | .986 | 1.375 |
|  | [ATdesin=5] | 0^b^ | . | . | 0 | . | . | . | . |
|  | [AThipertim=1] | -.021 | .137 | .023 | 1 | .880 | .980 | .749 | 1.281 |
|  | [AThipertim=2] | -.027 | .137 | .040 | 1 | .842 | .973 | .744 | 1.273 |
|  | [AThipertim=3] | .067 | .137 | .242 | 1 | .623 | 1.070 | .818 | 1.400 |
|  | [AThipertim=4] | .164 | .140 | 1.367 | 1 | .242 | 1.178 | .895 | 1.552 |
|  | [AThipertim=5] | 0^b^ | . | . | 0 | . | . | . | . |
|  | [ATCeuf=1] | .152 | .102 | 2.203 | 1 | .138 | 1.164 | .952 | 1.423 |
|  | [ATCeuf=2] | .061 | .104 | .345 | 1 | .557 | 1.063 | .867 | 1.303 |
|  | [ATCeuf=3] | .076 | .098 | .613 | 1 | .434 | 1.079 | .891 | 1.307 |
|  | [ATCeuf=4] | .071 | .090 | .613 | 1 | .434 | 1.073 | .899 | 1.281 |
|  | [ATCeuf=5] | 0^b^ | . | . | 0 | . | . | . | . |
|  | [sex=1] | .395 | .110 | 12.849 | 1 | .000 | 1.485 | 1.196 | 1.843 |
|  | [sex=2] | 0^b^ | . | . | 0 | . | . | . | . |

ATdep: Affective temperament depressive; ATans: Affective temperament avoidant; ATapat: Affective temperament apathetic; ATobses: Affective temperament obsessive; ATciclo: Affective temperament cyclothymic; ATdisf: Affective temperament dysphoric; ATeut: Affective temperament euthymic; ATirrit: Affective temperament irritable; ATvolat: Affective temperament volatile; ATdesin: Affective temperament disinhibited; AThipertim: Affective temperament hyperthymic; ATCeuf: Affective temperament euphoric; Sex=1: Men; Sex=2: Women.
